# Eye-tracking paradigms for the assessment of mild cognitive impairment: a systematic review

**DOI:** 10.3389/fpsyg.2023.1197567

**Published:** 2023-07-20

**Authors:** Alexandra Wolf, Kornkanok Tripanpitak, Satoshi Umeda, Mihoko Otake-Matsuura

**Affiliations:** ^1^Cognitive Behavioral Assistive Technology (CBAT), Goal-Oriented Technology Group, RIKEN Center for Advanced Intelligence Project (AIP), Tokyo, Japan; ^2^Department of Neuropsychiatry, Graduate School of Medical Sciences, Kyushu University, Fukuoka, Japan; ^3^Department of Psychology, Keio University, Tokyo, Japan

**Keywords:** Alzheimer’s disease, biomarker, dementia, eye-tracking, cognitive assessment, information processing, mild cognitive impairment, screening

## Abstract

Mild cognitive impairment (MCI), representing the ‘transitional zone’ between normal cognition and dementia, has become a novel topic in clinical research. Although early detection is crucial, it remains logistically challenging at the same time. While traditional pen-and-paper tests require in-depth training to ensure standardized administration and accurate interpretation of findings, significant technological advancements are leading to the development of procedures for the early detection of Alzheimer’s disease (AD) and facilitating the diagnostic process. Some of the diagnostic protocols, however, show significant limitations that hamper their widespread adoption. Concerns about the social and economic implications of the increasing incidence of AD underline the need for reliable, non-invasive, cost-effective, and timely cognitive scoring methodologies. For instance, modern clinical studies report significant oculomotor impairments among patients with MCI, who perform poorly in visual paired-comparison tasks by ascribing less attentional resources to novel stimuli. To accelerate the Global Action Plan on the Public Health Response to Dementia 2017–2025, this work provides an overview of research on saccadic and exploratory eye-movement deficits among older adults with MCI. The review protocol was drafted based on the Preferred Reporting Items for Systematic Reviews and Meta-Analyses guidelines. Electronic databases were systematically searched to identify peer-reviewed articles published between 2017 and 2022 that examined visual processing in older adults with MCI and reported gaze parameters as potential biomarkers. Moreover, following the contemporary trend for remote healthcare technologies, we reviewed studies that implemented non-commercial eye-tracking instrumentation in order to detect information processing impairments among the MCI population. Based on the gathered literature, eye-tracking-based paradigms may ameliorate the screening limitations of traditional cognitive assessments and contribute to early AD detection. However, in order to translate the findings pertaining to abnormal gaze behavior into clinical applications, it is imperative to conduct longitudinal investigations in both laboratory-based and ecologically valid settings.

“Dementia research needs to be conducted within an enabling environment where collaborations are fostered, and equitable and sustained investment is realized (WHO, 2022).”

## 1. Introduction

**Figure 1:**
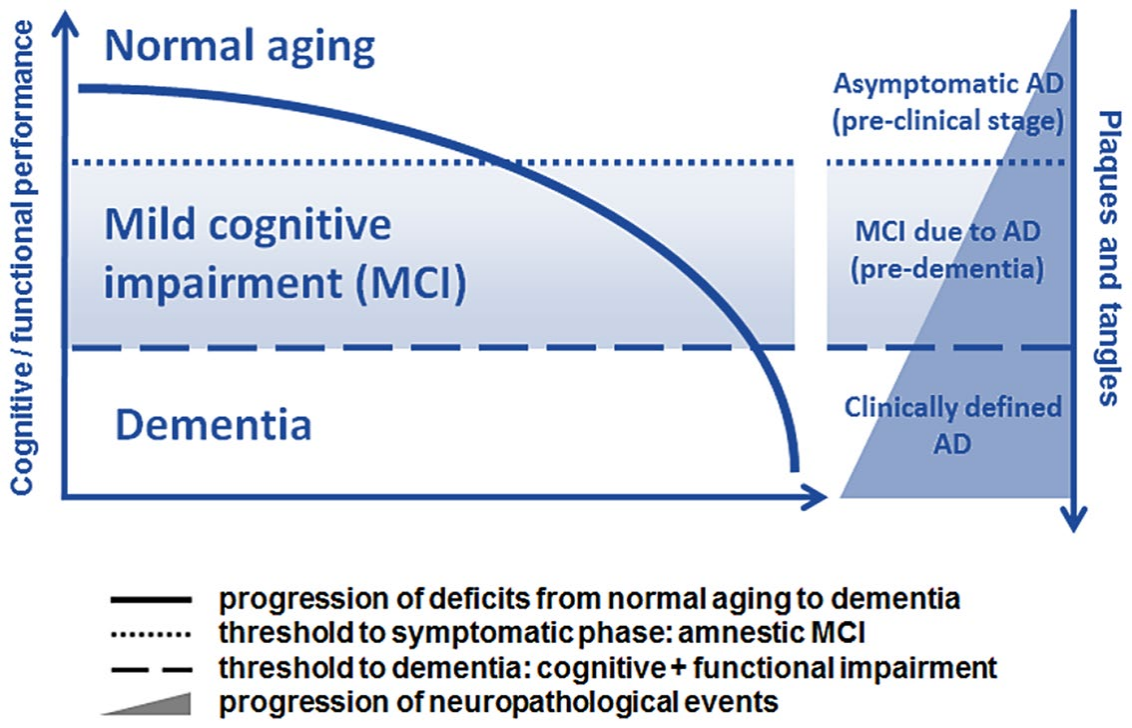
Schematic progression from normal aging to dementia, adapted with modifications in color from Forlenza et al., 2010 (available *via* license: CC BY 2.0). Relationship between the progression of cognitive and functional symptoms and the neuropathological events in the transition from the pre-clinical (silent) phase to mild cognitive impairment (MCI) due to AD and clinically manifest AD.

The pathology of Alzheimer’s disease (AD) may begin up to 20 years prior to the onset of severely debilitating symptoms (Jack et al., 2011). While potentially disease-modifying cognitive intervention therapies are being intensively developed, there is a need for sensitive and readily available screening tools that can detect AD in its initial stages (Otake-Matsuura et al., 2021). Mild cognitive impairment (MCI) is a term used to describe the transitional phase between the average cognitive decline that comes with normal aging and the onset of major neurocognitive disorder (commonly referred to as ‘*dementia*’; Petersen et al., 1999; Bruscoli and Lovestone, 2004; Roberts and Knopman, 2013; Kasper et al., 2020; Sabbagh et al., 2020a). Simply put, MCI can be portrayed as an early window for detecting cognitive impairment prior to the progression of neurodegenerative disease (see Figure 1; Roberts and Knopman, 2013; Ataollahi Eshkoor et al., 2015; Dunne et al., 2021). Neuropsychological symptoms may be absent during the latent phase, despite the presence of neuropathologic changes (including neurotic plaques and neurofibrillary tangles) that are primarily related to the overproduction and aggregation of amyloid beta (Aβ) peptide within the brain and to the hyperphosphorylation of Tau protein in affected neurons (Forlenza et al., 2010). As the pathology progresses, cognitive deterioration, such as worsening memory problems, poor judgment, confusion, difficulty in speaking, understanding, and expressing thoughts or reading and writing, begins to surface (*prodromal stage*). If not identified and addressed, a fully manifested clinical disease with irreversible consequences to one’s daily living abilities may develop (Alzheimer’s Association, 2019). Research has shown that after approximately 6 years, 80% of individuals with MCI progress to dementia (Petersen, 2003; Busse et al., 2006).

Furthermore, MCI is characterized by different subtypes, including amnestic MCI (aMCI), single-domain non-amnestic MCI (naMCI), and multiple-domain MCI. It has been postulated that the amnestic type presents itself predominantly with memory impairment (Kawagoe et al., 2017). Notably, although memory has been reported to be negatively affected in aMCI (Kahana Levy et al., 2018), impairments in other cognitive domains, such as executive function and visuospatial ability, may remain dormant if they do not affect the individual’s activities of daily living (Gold and Budson, 2008; Johnson et al., 2009; Niu et al., 2013); hence, older adults may not complain about them (Kawagoe et al., 2017). The non-amnestic form of MCI, on the other hand, is reportedly accompanied by deficits in cognition and motor performance (with preserved memory; Petersen et al., 1999; Kluger et al., 2008; Readman et al., 2021). Since memory loss and cognitive decline occur in multiple-domain MCI (Kramer et al., 2006; Ataollahi Eshkoor et al., 2015), amnestic and multiple-domain MCI subtypes have been proposed to pose an equal risk for Alzheimer’s disease (AD) progression (Petersen et al., 1999; Gauthier et al., 2006; Fischer et al., 2007; Ward et al., 2013; Ataollahi Eshkoor et al., 2015; Dunne et al., 2021). Notwithstanding, it has been suggested that the classification of aMCI as specific to AD and naMCI to other dementias (particularly vascular dementia) is *“conceptually too simplistic”* (Busse et al., 2006; Albert et al., 2007; Fischer et al., 2007; Rosenberg and Lyketsos, 2008). However, independent research groups exploring the structural differences between various MCI forms have provided scientific evidence to support the notion that separating these subtypes is not only a theoretical concept. For example, structural imaging and neuropsychological testing has supported the distinction between amnesic and non-amnesic forms of MCI. In the context of non-brain measures, such as eye-tracking, individuals with aMCI were found to be less accurate than controls and individuals with naMCI while performing a recognition task (McCade et al., 2018). Moreover, significant differences between aMCI and naMCI are highlighted by divergence in the percentage of uncorrected errors in the anti-saccade task (Wilcockson et al., 2019; Koçoğlu et al., 2021).

A variety of visual problems have been reported in patients with AD, including loss of visual acuity, abnormalities in contrast sensitivity, defects in fixation and saccadic eye movements, and disturbances of complex visual functions such as reading, naming, and identifying objects (Armstrong, 2009). Therefore, since visual cognitive dysfunctions transpire as an early indication of the transition from MCI to AD (Nakashima et al., 2010; Polden et al., 2020; Wolf and Ueda, 2021; Hannonen et al., 2022), visual testing holds promise for facilitating clinical diagnosis in future scenarios (Crutcher et al., 2009; Haque et al., 2019; Oyama et al., 2019; Readman et al., 2021; Tadokoro et al., 2021). Furthermore, and crucially, a deeper understanding of MCI subtypes may aid in predicting progression to AD and facilitate the development of targeted prevention strategies (Csukly et al., 2016; Kahana Levy et al., 2018; Opwonya et al., 2022b).

The problem of controlling AD-related healthcare costs while advancing health equity and quality has become an increasingly urgent issue to address (Pereira et al., 2020; Cilia et al., 2022; Kharroubi and Elbarazi, 2023). To visualize the pressing situation, in 2012, a new case of dementia was diagnosed every 7 s (Rashid et al., 2012), but more recent data indicate that every 3 s, someone in the World develops dementia (Alzheimer’s Association, 2019). In addition, while significant efforts are being devoted to discover drugs to slow down the progression of AD or alleviate its symptoms, few are authorized for clinical use (Ishikawa et al., 2022). Simultaneously, despite the vast research on AD, no single assessment measure is capable of predicting the onset of AD in a non-invasive, timely, and cost-effective manner (Bruscoli and Lovestone, 2004; Petersen, 2004; Panza et al., 2005; Zola et al., 2013; Ishikawa et al., 2022). Accordingly, clinicians are left with an arduous dementia diagnostic process based on a combination of laboratory tests, neuroimaging studies, and neuropsychological evaluations, which can take several months to complete (Petersen, 2003, 2004; Roberts and Knopman, 2013; Langa and Burke, 2019; Chen et al., 2021).

### 1.1. Eye-tracking as a potential solution to the challenges associated with assessment in MCI

According to the World Health Organization’s first blueprint for dementia research: *“(…) addressing dementia comprehensively requires research and innovation to be an integral part of the response”* (WHO, 2022). Undoubtedly, there is a need for far-reaching and cost-effective innovations that reliably support the process of MCI diagnosis and facilitate the early application of cognitive interventions (Sabbagh et al., 2020b). With advances in eye-tracking technology and results from scientifically backed paradigms, health professionals may receive practical and effective screening tools for AD-related MCI in the future (Oyama et al., 2019; Wolf and Ueda, 2021). Eye-tracking technology provides a promising foundation for future cognitive assessment protocols (Hanazuka et al., 2021; Ehrlich et al., 2022) and carefully selected gaze parameters could accurately reflect changes in cerebral physiology (Leigh and Zee, 2015), reducing the risk of incorrect diagnoses (Samadani et al., 2015; Samadani, 2016).

In psychiatry research, gaze parameters have been shown to be promising biomarkers of diseases such as depression, bipolar disorder, and schizophrenia (Wolf et al., 2021a). Recently, eye-tracking has gained scientific attention as a potential technology to facilitate the diagnosis and management of AD-related MCI (Seligman and Giovannetti, 2015; Oyama et al., 2019; Ołownia et al., 2021; Wolf and Ueda, 2021). Notably, by mirroring thought processes, gaze can expose early cognitive impairments (Polden et al., 2020; Wolf and Ueda, 2021). A recent meta-analysis performed by Liu and colleagues showed that eye-tracking technology can detect a decline in patients’ cognition (Liu et al., 2021). Concurrently, the passive monitoring of daily activity *via* smartphones, tablets, or smart-home devices provides portable means of tracking behavioral changes over time (Cichocki et al., 2008; Vashist et al., 2014; Miyake et al., 2020; Thabtah et al., 2020; Valliappan et al., 2020; Rutkowski et al., 2021; Wolf et al., 2021b). Following the digital healthcare trend, detecting cognitive deviations from the trajectory of normal aging through remote (non-face-to-face) channels has gained increasing interest (Rabinowitz and Lavner, 2014; Dagum, 2018; Huang et al., 2019; Kourtis et al., 2019). Eye-tracking technology represents a creative implementation of smart technologies that may support unsupervised at-home testing of cognitive performance (Dodge et al., 2015; Jekel et al., 2016; Rutkowski et al., 2020; Sabbagh et al., 2020a). Furthermore, advanced phone cameras combined with machine learning algorithms could support smartphone eye-tracking technology (Kong et al., 2021). Front-facing “selfie” cameras are particularly convenient for monitoring the performance of eye-movement tests on a more casual basis (Valliappan et al., 2020). Technological advances open up the possibility of particular gaze metrics being extracted from individuals while they perform experiments in front of a tablet or phone screen, contributing to a digital biomarker arsenal for disease detection (Kourtis et al., 2019; Kröger et al., 2020).

In recent years, the scientific literature has mounted in eye-tracking-based paradigms that aim to (i) gain insight into the visual abnormalities among cognitively unimpaired older adults, and (ii) improve the assessment of cognitive impairment due to AD. Hence, to accelerate the transition toward a globally accessible screening procedure for MCI (Sabbagh et al., 2020c, 2022; Liss et al., 2021), recent studies evaluating the potential utility of gaze metrics in the detection and characterization of MCI have been reviewed and discussed. Considering the multiple advantages of eye-tracking technology, it is hoped that presented compilation of impactful studies presented here, will spark interest among clinicians and foster future collaborations between neuroscience and machine learning, leading to an improved characterization of individuals along the Alzheimer’s disease trajectory (Lagun et al., 2011; Zola et al., 2013; Wolf et al., 2021a; Ning et al., 2022; Przybyszewski et al., 2023).

## 2. Methods

This systematic review aimed to identify studies of MCI-related gaze behavior impairments published in the past 6 years (2017–2022). The protocol was drafted based on the Preferred Reporting Items for Systematic Reviews and Meta-Analyses (PRISMA) guidelines (Page et al., 2021). Electronic databases (Edith Cowan University Library, PubMed, Semantic Scholar, and Springer) were systematically searched to identify peer-reviewed literature that examined visual processing among older adults, as well as studies comparing cognitively unimpaired individuals to elderly individuals with MCI. Studies were found using a combination of the following terms: “mild cognitive impairment” or “MCI” AND “diagnosis” or “screening” AND “biomarker.” Notably, the search term “eye-tracking” or “eye movements” were added to narrow the result to journal articles that reported gaze parameters as potential biomarkers for MCI. The search results (.csv file) obtained from each database were consolidated and saved as a single Microsoft Excel spreadsheet (.xls file). The spreadsheet was meticulously scrutinized for duplications through a manual inspection, which was carried out separately by AW and KT. Any disagreement was resolved by discussion and consensus. Certainly, following the preferred reporting items for PRISMA systematic review guidelines (Page et al., 2021), specific inclusion criteria were applied. To be included in this review, studies had to be relevant, original, peer-reviewed, and written in English. Furthermore, the studies had to include an MCI group (without comorbidities or other neurological disorders), which had to be evaluated by standardized diagnostic criteria and diagnosed with validated cognitive tests. Conference papers, letters, books, single case studies with a small sample (i.e., studies with less than 10 participants in the MCI and/or control group), and non-primary literature such as systematic reviews, meta-analyses, and editorials were excluded.

**Figure 2:**
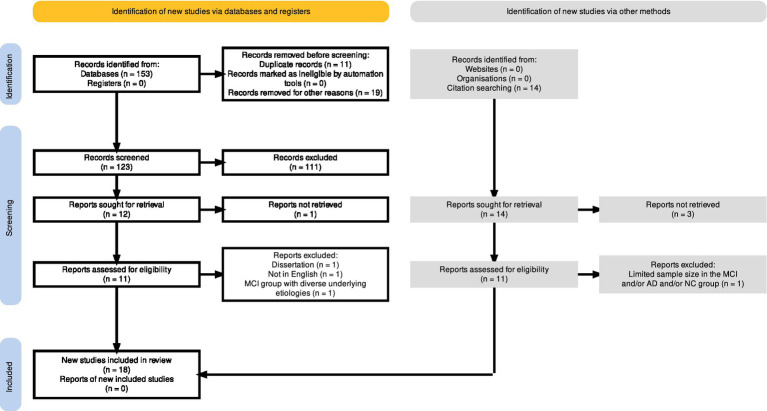
The full output plot from the PRISMA flow diagram, generated *via* the https://estech.shinyapps.io/prisma_flowdiagram/ (Haddaway et al., 2022).

The PRISMA flow diagram, depicted in Figure 2, was generated using a web-based and free-to-use Shiny app (Haddaway et al., 2022), which allows users to create customized PRISMA flow diagrams for their systematic reviews. Out of the one-hundred fifty-three initially identified records (*n* = 153), a total of eleven duplicates were detected and consequently eliminated prior to the screening process. Furthermore, among the identified records, eighteen (*n* = 18) entries were excluded for varying reasons, including the classification of eighteen positions as conference proceedings and/or abstract book titles, while one entry (*n* = 1) lacked an available abstract. Next, the screening process involved reviewing the titles and abstracts of one-hundred twenty-three (*n* = 123) records. Out of these, fifty-five studies were deemed irrelevant to mild cognitive impairment (MCI) or focused on different clinical conditions, such as Autism Spectrum disorder, Parkinson’s, schizophrenia, neurodevelopmental disorder or eating disorder. Additionally, four in-scope systematic reviews, two book chapters, and one study identified as a conference abstract, were rejected. Furthermore, the exclusion of forty-eight studies that examined various approaches for dementia screening was justified since these reports did not incorporate the use of eye-tracking technology. Also, one study focusing on the efficacy of a drug in enhancing visuospatial abilities among MCI patients through eye-tracking measurements was excluded. As a result, a total of one hundred and eleven records were excluded from the analysis due to their failure to meet the predetermined inclusion criteria. Next, a comprehensive search was undertaken to obtain twelve specific reports in the form of full-text papers. Out of the desired reports, eleven were successfully retrieved and checked for eligibility. Among the eleven reports, three were excluded (refer to the PRISMA flow diagram in Figure 2 for detailed reasons), resulting in the inclusion of eight reports (Oyama et al., 2019; Wilcockson et al., 2019; Nie et al., 2020; Gills et al., 2021; Haque et al., 2021; Chehrehnegar et al., 2022; Hannonen et al., 2022; Opwonya et al., 2022b). Notably, to supplement the identification of relevant studies, the reference lists of eight in-scope and full-text articles were independently screened by AW and KT for relevant publications. This practice, which is recommended in systematic review manuals (Horsley et al., 2011), served as an effective approach. In result, fourteen relevant studies for the systematic review have been identified. Eleven positions have been successfully retrieved as full-text documents for assessment of eligibility. After a detailed examination of the gathered works, one study was excluded due to the limited sample size in the MCI group (*n* < 10). Overall, the search of the reference lists has resulted in the addition of ten new studies (Galetta et al., 2017; Kawagoe et al., 2017; Bott et al., 2018; Noiret et al., 2018; Chehrehnegar et al., 2019; Gills et al., 2019; Haque et al., 2019; Pereira et al., 2020; Koçoğlu et al., 2021; Tadokoro et al., 2021).

In essence, this work presents a comprehensive review of the included studies, providing a thorough examination of the evidence on whether gaze metrics from eye-movement paradigms can distinguish between older adults with MCI, including those with the highest conversion rate to AD (aMCI subtype), and their age-matched counterparts. To combine the rising trend of eye-tracking technology with the challenges of AD diagnosis, the significant constraints of the currently used “ruling out” protocol have been elucidated. The research synthesis follows with an introduction of the human retina, capable of mirroring brain structure and revealing cognitive disturbances through human eye movements. Notably, the authors outline the fundamental point of gaze behavior as a reflection of one’s attention and thought processes. A straightforward follow-up statement is presented on why eye-tracking should be considered an attractive technology for facilitating a non-invasive diagnosis of MCI by providing meaningful and objective outcome measures. Notably, this work highlights eye movement tests that provide information about saccadic and exploratory impairments among the elderly population with MCI. Furthermore, specific eye-movement parameters, which show potential in distinguishing between patients with MCI and cognitively unimpaired elderly, have been identified.

## 3. “Ruling out” approach: the challenge of an early and accurate diagnosis

MCI is heterogeneous in its clinical spectrum (Kramer et al., 2006); therefore, this intermediate state is challenging to identify in clinical practice. Since some degree of cognitive slowing is typical in the context of healthy aging, identifying clinically significant cognitive impairments remains clinician’s primary challenge (Hugo and Ganguli, 2014). An early and accurate diagnosis may give a patient the chance for improved quality of life and preserved independence in activities of daily living (Seligman and Giovannetti, 2015; Davis et al., 2018; Kasper et al., 2020; Budson and Solomon, 2021). However, there is a reported lack of technical support, infrastructure, training, and experience among primary care physicians to efficiently detect preclinical phases and manage AD along its clinical continuum (Olazaran et al., 2011; Kasper et al., 2020; Sabbagh et al., 2020b,c). For instance, a survey conducted in the United States revealed that only half of adults aged above 65 years undergo cognitive evaluations. This significant finding has been attributed to factors such as time constraints, the subtlety of patients’ cognitive impairment, and resistance from elderly individuals towards being tested (Alzheimer’s Association, 2019). Since the role of primary care physicians, being the first medical professionals that patients reach out to, is vital in the identification and management of MCI (Olazaran et al., 2011; Sabbagh et al., 2020c), rapid routine recordings of eye movements in the primary care setting could provide an objective and time-efficient method to facilitate diagnosis.

The necessity for a sharp demarcation between normal cognition and MCI as well as between MCI and AD remains crucial (Albert et al., 2011; Sabbagh et al., 2020b). To make these distinctions, several findings and clinical judgments must be integrated and interpreted. Extensive neuropsychological cognitive screening tests such as the Montreal Cognitive Assessment (MoCA), the Alzheimer’s Disease Assessment Scale-Cognitive Subscale (ADAS-Cog), Cognistat (formerly known as the Neurobehavioral Cognitive Status Examination), and the short Mini-Mental State Examination (MMSE) can be incorporated into the preliminary assessment (refer to Breton et al., 2019 for an insightful meta-analysis of diagnostic accuracy studies). These pen-and-paper tests contain elements related to executive functions, memory, orientation, learning, judgment, and perceptual motor function, and are commonly used in the clinical setting (Folstein et al., 1975; Bobholz and Brandt, 1993; Hanazuka et al., 2021). Furthermore, to evaluate verbal memory, two specific tests (the Rey Auditory Verbal Learning Test and Wechsler Memory Scale—IV—Logical Memory subset) may have utility during neuropsychological assessment (Rabin et al., 2005). Last but not least, the currently employed diagnostic protocols may require older adults to undergo a depression screening, since mood disorders can also cause dementia-like symptoms, including memory problems and a loss of interest in life (Dierckx et al., 2007, Defrancesco et al., 2009; for a review of putative neuropsychological mechanisms leading from depression to the development of AD, see Tetsuka, 2021).

In theory, a subject’s score (performance) on a test is compared to a large general population normative sample derived from a population comparable to the person being examined. Based on this comparison, one’s most recent cognitive functioning can be evaluated (Grossman et al., 1996; Hansen et al., 2018; Dunne et al., 2021). Nonetheless, despite being considered cost-effective and straightforward to administer, cognitive function tests are not sufficiently sensitive to identify the progression of MCI (for example, ADAS-Cog may be less responsive to change when used in people with MCI; Skinner et al., 2012; Thabtah et al., 2022b). Notably, as writing and drawing are required in some tests, motor impairments such as post-stroke paralysis (frequently observed in patients with dementia) can lead to lower scores and inaccurate diagnoses (Palsetia et al., 2018; Heyrani et al., 2022). Other factors that could potentially influence screening results have been discussed in the literature, such as the experience and training of the examining clinician as well as a potential dependency on the used screening test (Hoops et al., 2009). In addition, a further potentially confounding factor is the lack of a clear collateral history regarding prior peak occupational or educational attainments. Thus, relying on the neuropsychological score makes it challenging to detect MCI among high-functioning older adults (Tuokko et al., 2003; Dunne et al., 2021), where, simply speaking, impaired cognitive functioning in these individuals may not come to medical attention (Treves et al., 2005; Chary et al., 2013; Jessen et al., 2014; Dunne et al., 2021).

Patient evaluations remain challenging (Roberts and Knopman, 2013; Jekel et al., 2016; Oyama et al., 2019; Kasper et al., 2020) especially when taking into consideration that patients may (i) face problems with language comprehension or articulation while talking with healthcare professionals, (ii) experience high levels of psychological stress and fatigue while answering a series of questions during the assessment, or (iii) not have an accurate understanding of their own cognitive capabilities (Grossman et al., 1996; Gates et al., 2002; Hanazuka et al., 2021). Taken together, although neuropsychological screenings are still considered helpful in assessing respondents’ cognitive functions, they are far from being objective.

Although this review does not aim to list all the advantages and shortcomings of the currently applied ‘traditional pen-and paper’ tests, note that inherent drawbacks of such tools have led to a concerted research effort to identify alternate diagnostic methods (Sonnen et al., 2008; Sabbagh et al., 2020b; Chen et al., 2021; Ning et al., 2022). For example, to confirm AD physicians may use a variety of approaches and tools, including blood and cerebrospinal fluid (CSF) biomarkers (Galasko, 2015; Hameed et al., 2020). Moreover, besides undergoing physically invasive assessments such as lumbar punctures, other intensive neuroimaging techniques including magnetic resonance imaging (MRI) are widely used to investigate brain changes (for example, cortical thickness) due to neurodegeneration (Raamana et al., 2014). Finally, diffusion tension imaging (DTI), positron emission tomography (PET), and proton magnetic resonance spectroscopy (^1^H-MRS) are being investigated to define the biological AD construct (Jack et al., 2018). However, although PET is reportedly successful in characterizing cerebral Aβ plaques (Jansen et al., 2015), this particular technique is considered invasive, costly, and inaccessible; hence, it is unsuitable for population-based AD screening (Koronyo et al., 2017; Yang et al., 2019; Wang and Mao, 2021).

Overall, despite significant research efforts to acquire an early and more accurate AD diagnosis, the call for action to address the social and economic consequences of major neurocognitive disorders persists. AD remains incurable (Soleimani Zakeri et al., 2020), which increases the urgency for action. Moreover, although the Global Action Plan on the Public Health Response to Dementia 2017–2025 has been put in place (WHO, 2017), the majority of countries are yet to achieve the targets set in the plan (Werner, 2012; Lin and Neumann, 2013; Casagrande et al., 2022; see Global Status Report on the Public Health Response to Dementia, WHO, 2021). While policymakers around the world emphasize the importance of developing a successful diagnostic protocol, the authors would like to emphasize eye-tracking technology as a non-invasive, cost-effective, sensitive, and convenient response to the global call for action in addressing the extraordinary burden of AD (Wright and O’Connor, 2018; Tahami Monfared et al., 2022). Considering the fact that the retina is an optically accessible developmental outgrowth of the central nervous system (Eckstein et al., 2017), it has been postulated that changes in one’s eye could reflect pathological processes occurring within the brain (Armstrong, 2009; Kumar et al., 2015; Nguyen et al., 2021; Wang and Mao, 2021; Wolf et al., 2021a). As a result, researchers seeking to distinguish between healthy and pathological aging have, in recent years, turned to the human eye (Criscuolo et al., 2018; Ramzaoui et al., 2018; Mirzaei et al., 2020; Hanazuka et al., 2021; Nguyen et al., 2021; Wolf and Ueda, 2021; Romaus-Sanjurjo et al., 2022).

## 4. The eye: anatomical extension of the brain

Although ancient scholars crowned the eyes with the title of the *windows to one’s mind*, modern ocular-neural imaging techniques have scientifically confirmed that several well-defined neurodegenerative conditions as well as psychiatric disorders manifest themselves in the detailed structure of the human eye (Santos et al., 2018; Majeed et al., 2021). Furthermore, the fact that both the eye and the brain “*modify similarly with disease*” (Nguyen et al., 2021) creates a rich research opportunity. Hence, it stands to reason that investigating the human eye mirroring pathological processes that occur in the brain will become a rapidly expanding field of research. Recent ocular imaging studies, including methods such as optical coherence tomography (OCT) and optical coherence tomography angiography (OCTA), have indicated that AD is associated with a decreased volume of the optic nerve, degeneration of retinal ganglion cells, loss in retinal nerve fiber layer (RNFL), and deposition of abnormally structured proteins (de Oliveira et al., 2020). Following the conclusion that the eye’s microarchitecture is profoundly affected by AD and has the potential to harbor the earliest detectable disease-specific signs, the development of optical biomarkers for AD and other neurodegenerative disorders has gained significant interest in the context of clinical applications (for a comprehensive review on ocular biomarkers for AD diagnostics, readers are encouraged to read the work of Majeed et al., 2021).

Independent research groups have found a significant reduction in RNFL layer thickness in individuals with AD compared to cognitively unimpaired healthy controls (Garcia-Martin et al., 2014; Santos et al., 2018; Alber et al., 2020; Majeed et al., 2021). In parallel, this structural change has also been associated with Lewy body dementia, Parkinson’s disease, multiple sclerosis, and conditions such as stroke and late-life depression. Therefore, it has been postulated that RNFL thinning alone is insufficient for a diagnosis of AD (Snyder et al., 2021) and – for the current state of knowledge – may only be a useful biomarker for a broader diagnosis of neurological pathologies (Ngolab et al., 2019). Additionally, it has been reported that ocular diseases such as glaucoma and non-glaucomatous optic neuropathology can also lead to pathological changes in the retina, making it challenging to develop clinically validated ocular biomarkers for AD. Some preliminary evidence suggests that Aβ deposits in the retina appear to be specific to patients with AD (Bilgel et al., 2016; Koronyo et al., 2017; Hadoux et al., 2019; Dumitrascu et al., 2020). However, the results of investigations that directly targeted Aβ accumulations were limited, leaving the scientific community with practically no clinically validated ocular biomarkers for AD (Wang and Mao, 2021).

The lack of sensitive and specific OCT/OCTA parameters as well as standardized imaging protocols (affecting the variability of structural markers) have been explicitly underlined in the scientific literature. Mentioned limitations hamper the use of ocular structures as influential and cost-effective biomarkers (Lee et al., 2020; Majeed et al., 2021). Moreover, the advice of using optical tomography in accordance with another technique such as MRI or biochemical analyses (Hashmi and Muzzammel, 2020) not only prolongs the diagnostic process, but also increases the number of involved medical doctors such as geriatricians, ophthalmologists, neurologists, and radiologists (Liss et al., 2021). This, in turn, generates high personnel- and equipment-related costs.

Eye-tracking devices, on the other hand, are regarded as relatively low-cost assessment tools, requiring only the presence of a technician who can be trained to explain and carry out the test. Moreover, the location of data collection can be extraordinarily flexible and take place in any comfortable environment, not restricted to the surroundings of a hospital, which is usually the case with neuroimaging apparatus. In addition, since most eye-tracking-based paradigms do not require verbal responses, scientists find gaze parameters extremely useful in assessing cognitive capacities among patients with language comprehension problems (Readman et al., 2021).

## 5. Objective

The utility of eye-tracking technology is receiving great interest in distinguishing people with neurocognitive disorders from their healthy counterparts (Anderson and MacAskill, 2013; Eckstein et al., 2017; Liu et al., 2021; Wolf and Ueda, 2021; Opwonya et al., 2022b). The concept is simple, and core brain damage associated with AD does not have to be directly evaluated through extensive physical assessments involving visualizations of the human eye or brain. Significant physiological changes, such as the accumulation of the pathological hallmarks of AD (intracellular neurofibrillary tangles, senile plaques), and the subsequent disruptions in synaptic transmission result in profound cognitive impairments (Baddeley, 2001; Forlenza et al., 2010; Kumar et al., 2015; Readman et al., 2021). Current evidence suggests that attention is the initial non-memory domain to be affected in AD, with visual information processing impairments occurring in the MCI phase (Ramzaoui et al., 2018; Polden and Crawford, 2021; Readman et al., 2021). As attention and oculomotor control are thought to recruit overlapping brain regions, saccades (for example) are likely to be disturbed by the reductions in inhibitory control and executive function that occur in neurodegenerative disorders (Wollenberg et al., 2018).

In the light of a noticeable shift in focus to context-processing impairments and cognitive remediation for addressing cognitive impairments, the study of saccadic abnormalities and impairments in visual information processing has become a high-priority research area (Wolf and Ueda, 2021; Kim et al., 2022). Trends in eye-tracking assessment align well with evidence that human gaze gives powerful insights regarding one’s information processing patterns (Eckstein et al., 2017; Marandi and Gazerani, 2019; Kröger et al., 2020; Nie et al., 2020; Chehrehnegar et al., 2022; Opwonya et al., 2022b). This opens new opportunities to provide proxy instrumentation to measure cognition (and its deficits) and disclose hidden aspects of aging (Molitor et al., 2015; Marandi and Gazerani, 2019). Therefore, apart from quantifying the parameters of an effectively stabilized (*frozen in time*) retina, scientists have begun to mirror the observer’s brain integrity of sensory function and predict disease processes (Samadani et al., 2015; Lauermann et al., 2017; Marandi and Gazerani, 2019; Snyder et al., 2021; Wolf et al., 2021a; Opwonya et al., 2022b).

Undoubtedly, the scientific community requires more profound information regarding gaze metrics obtained from experimental paradigms that include older adults. While the next decade of clinical research is likely to lead to gaze parameters being included in clinical cognitive testing (Crutcher et al., 2009; Bott et al., 2017; Gills et al., 2019, 2021; Oyama et al., 2019; Tadokoro et al., 2021), the presented work introduces paradigms that incorporate eye-tracking technology into the challenging process of MCI assessment. These summarized insights from scientifically recognized and equally accessible protocols should support the future development of innovative response strategies and attenuate the dramatic financial burden of AD (Tarawneh and Holtzman, 2012; Klyucherev et al., 2022). Finally, the authors hope that the gathered evidence will spark interest among clinicians and foster cutting-edge, interdisciplinary collaborations to further research in this area.

## 6. Gaze: an indirect link to neural and cognitive functions

In recent years, to trace age-related irregularities associated with cognitive decline, researchers started to involve a variety of pupil-, fixation-, and saccade-related metrics serving as objective biomarkers (Marandi and Gazerani, 2019). Although human gaze is not a direct measure of their brain function, it does provide details on the association between the brain and behavior. Furthermore, in combination with attention-demanding tasks that demand one to act upon and manipulate given information, eye-tracking offers an interesting solution for future monitoring of the AD continuum (Ramzaoui et al., 2018; Opwonya et al., 2022a,b for scientific articles on bridging eye-tracking technology with cognitively informative paradigms and medical science, refer to works by Liu et al., 2021; Wolf and Ueda, 2021).

Yet, first and foremost, for an eye-tracking test to be an efficacious diagnostic tool, it must be able to differentiate those with preclinical cognitive decline (MCI) from cognitively unimpaired older adults as well as those with AD. It has been reported that changes in functioning of the frontal lobe and cingulate cortex can already lead to subtle impairments in inhibitory control. Since saccadic eye movements are primarily controlled by the frontal cortex, saccadic eye movements (SEM) have been suggested to offer important clues to facilitate the detection of the early signs of MCI. With this in mind, the authors hope that the referenced observations in the following section will be helpful to researchers and clinical practitioners who consider implementing saccade paradigms in order to expand the monitoring procedure of older adults at risk of MCI.

### 6.1. SEM impairments in MCI

**Figure 3:**
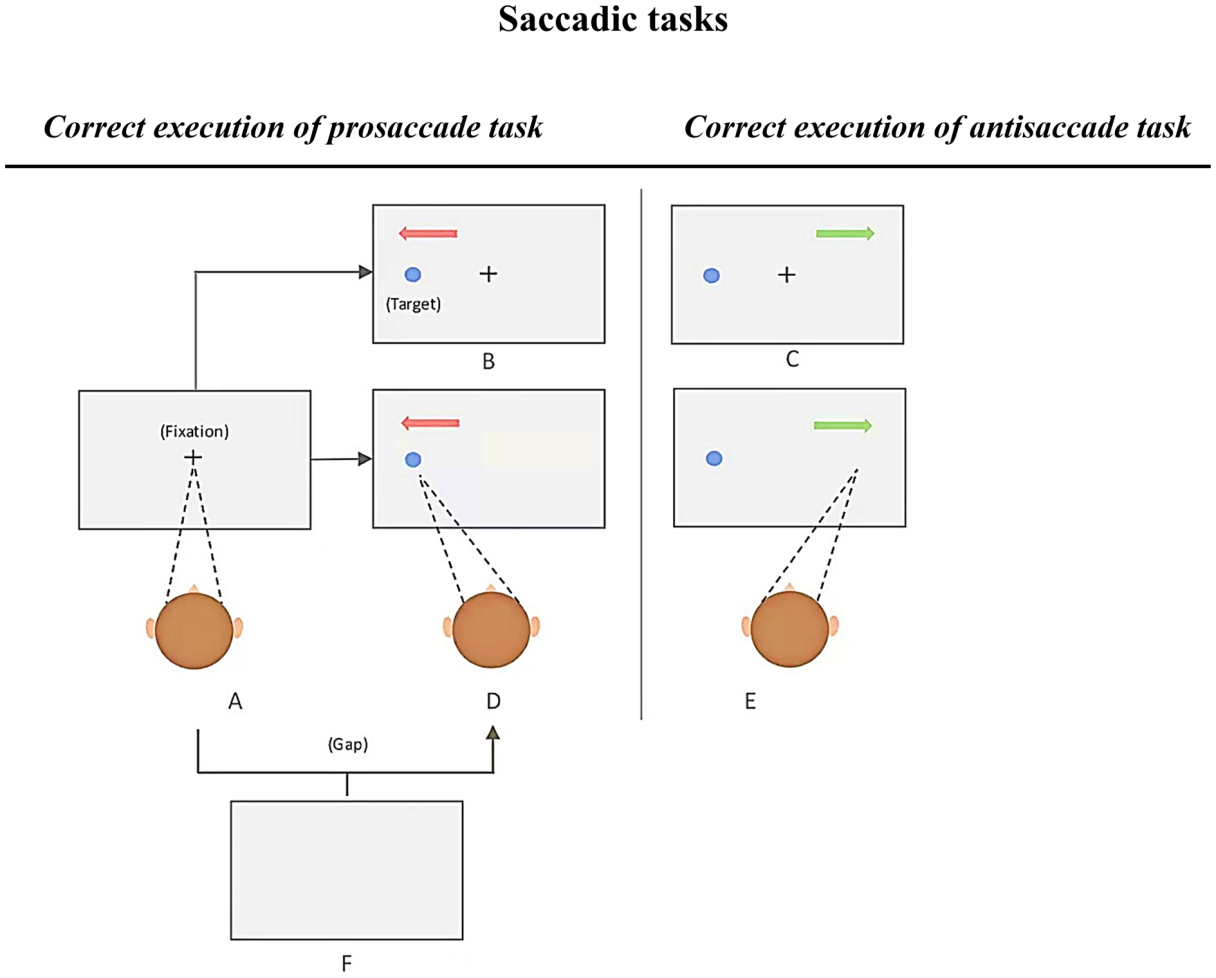
Various conditions of the prosaccade and antisaccade tasks, adapted from Si et al., 2022. Each trial begins with a presentation of a fixation cross at the center of the screen. Participants are required to fixate on it and to make a prosaccade or antisaccade (depending on the task’s instruction). Immediate prosaccade: A-D; Immediate antisaccade: A-E; Gap prosaccade: A-F-D; Gap antisaccade: A-F-E; Overlap prosaccade: A-B; Overlap antisaccade: A-C.

Previous literature outlined robust findings demonstrating saccadic abnormalities among patients with AD (Noiret et al., 2018). Most of these findings are relative to well-known prosaccade (PS) and antisaccade (AS) tasks. These tasks are particularly popular due to their potential measures of cognitive capacities as well as the simplicity of the instructions. In short, participants are requested to first keep their gaze on a central fixation, then, as quickly as possible, look at a target appearing at the periphery of the fixation marker (immediate PS, see Figure 3 [A–D]), or to direct their gaze to another direction, which is opposite to the target’s location (immediate AS, see Figure 3 [A–F]). A correct antisaccade performance consists of two main saccadic processes, namely, to restrain from making a saccade toward the target and voluntarily move the gaze in the opposite direction (Chehrehnegar et al., 2019; Si et al., 2022; Opwonya et al., 2022b). Hence, in the context of neurocognitive disorders such as AD, AS performance may reflect impairments in executive as well as attention functions, whereas PS performance may reflect the altered ability to rapidly trigger endogenous saccades toward a target, especially when viewer’s attention remains on the central fixation sign (overlap conditions, see in Figure 3; Noiret et al., 2018).

To investigate the diagnostic value of saccadic eye movements, Chehrehnegar and colleagues carried out PS and AS tasks and used two variants of saccade tasks, *gap* and *overlap*. In the *gap condition,* a black fixation cross was presented in the middle of the screen and randomly stayed on for 1,000 or 1,500 milliseconds. In the last 500 milliseconds, the fixation cross changed its color to green (PS task) or red (AS task). The fixation cross disappeared for a period of 200 milliseconds (hence, *gap condition*), and re-appeared along with the peripheral stimulus. In the *overlap condition* however, the fixation cross remained displayed for 200 milliseconds combined in time with the onset of the target stimulus. In both tasks, the target was randomly displayed at the left or right side of the fixation cross. Notably, this procedure required the participants to remember instructions in order to (in case of an AS, for example) inhibit the visually guided exogenous saccade toward the target, and to trigger a saccade in the opposite direction. Therefore, only when the instruction was correctly remembered, could the urge of making a reflexive response towards a target have been suppressed with a volitional saccade carried out in the opposite direction.

Concerning the available literature, a commonly used parameter in saccade-related paradigms is the *saccade latency*, which is the reaction time between the appearance of the target and the initiation of the orienting saccade. According to the results presented by Chehrehnegar et al. (2019), the reaction time was longer among participants with aMCI and AD when compared to healthy controls (HCs). The general increase in time of processing speed relates to increased motor and sensory processing times, which could be related to AD signatures in cortical regions. The observation of differences in saccadic reaction times between aMCI and HCs (Chehrehnegar et al., 2019) aligns with the suggestion that subjects with aMCI can be portrayed to be at an intermediate level of performance between HCs and patients with AD (Wilcockson et al., 2019; Pereira et al., 2020).

By examining another commonly used eye-movement parameter, the accuracy of a saccade (*saccade gain*), Chehrehnegar and colleagues identified this metric as the most sensitive measure to distinguish between individuals with aMCI and HCs (AS gap task, area under the curve [AUC] = 0.7; PS gap task, AUC = 0.63; AS overlap task, AUC = 0.73; the only paradigm that did not show any differences between aMCI and normal elderly was the overlap PS task). Moreover, since saccade gain was strongly correlated with neuropsychological measures, it has been speculated that this parameter could be of significant use to identify subtle executive deficits in the aMCI population. Thus, Chehrehnegar and colleagues highlighted that combining the antisaccade task with commonly used neuropsychological batteries may result in an improved sensitivity; for example, the use of the Addenbrookes Cognitive Examination in combination with the first gain parameter from the AS task resulted in an improved sensitivity index of 0.97.

Previous scientific contributions supported the notion that the AS task may be an additional prognostic tool that can differentiate the manifestations of preclinical cognitive decline. However, many of these studies referred to comparisons between patients with AD and healthy control groups. Therefore, further investigations that reveal saccadic impairments among elderlies with a higher risk for dementia due to AD (aMCI) would provide compelling support for the validity of the saccadic tasks as an early diagnostic marker.

With this objective in mind, in 2022 Chehrehnegar and colleagues performed another study that aimed to further investigate the possibility of distinguishing between HCs and participants with aMCI and AD. Several saccade parameters (including saccade amplitude and reaction time, error rates, omissions, and uncorrected saccades) were measured to clarify whether these biological markers are sensitive enough to clearly distinguish between healthy aging controls and cognitively impaired groups (MCI and AD). As in previous work, AS and PS tasks with *gap* and *overlap* conditions were implemented (Chehrehnegar et al., 2019). Notably, the researchers emphasized that after looking in the wrong direction, patients with aMCI had extreme difficulty in correcting their eye positions. Hence, when compared to HCs, the gaze behavior of the aMCI group was characterized by a greater number of errors and more saccade omissions (Chehrehnegar et al., 2022).

To elaborate more on the errors on the antisaccade task, they are most prevalent when the participants move their gaze toward the displayed target rather than away from it (also called the *error prosaccade*). The situation when participants make an error but quickly correct it, by looking away from the presented stimulus, is referred to as a self-corrected error. In a manner similar to patients with AD, older adults with MCI are prone to not correct committed errors due to alterations in the self-monitoring and correction network, which recruits the prefrontal cortex and anterior cingulate region. This result aligns with error monitoring and impairment of inhibitory control demonstrated by Wilcockson et al. (2019). They observed that the percentage of uncorrected AS errors of patients with AD and the amnestic variant of MCI was not only similar but also higher than subjects with naMCI and HC. Furthermore, in a more recent study, another independent research group noted greater failure to self-correct made mistakes among adults with aMCI, generating a high proportion of erroneous saccades (Opwonya et al., 2022b). Thus, an elevated error rate and abnormally high number of uncorrected saccades can be regarded as future markers for the early detection of aMCI (Peltsch et al., 2014) and mild AD (Opwonya et al., 2022a,b). On another note, in contrast to a previous report (Chehrehnegar et al., 2019), the follow-up study by Chehrehnegar and colleagues showed that the time to initiate saccades did not differ between subjects with aMCI and the HC group (Chehrehnegar et al., 2022). Given that saccadic reaction time may not be disrupted during the early stages of cognitive decline, the potential use of this particular gaze parameter remains debatable.

Although the clinical significance of saccadic eye movement impairments in MCI remains to be fully elucidated, researchers continue to search for alternative paradigms for discriminating between subtypes and assessing cognitive functioning among adults. A recent study performed by Koçoğlu and colleagues outlined differences in saccadic eye movements between the subtypes of MCI and HCs. While performing recordings of horizontal and vertical antisaccades, it was reported that, in comparison to HCs, patients with aMCI have a higher percentage of “express” saccades (defined as visually driven short latency saccades with response times falling between 80 and 120 milliseconds). Moreover, following the horizontal and vertical AS paradigm, the researchers reported a strong association between saccadic reaction time and participants’ cognitive status. The saccadic reaction time of corrected errors in the aMCI (*p* = 0.001) and naMCI (*p* = 0.038) groups were significantly longer than those in the HC group (Koçoğlu et al., 2021).

Next, following the context of alternative paradigms, it would be prudent to briefly mention the predictive saccades (PreS) task in which participants are instructed to direct their gaze in expectation of the emergence of a target in a particular spot with a fixed temporal frequency. Notably, in relation to current knowledge, this task has not been employed in research concerning the differentiation between MCI subtypes despite the notion that it could be used to reflect patients’ decreased ability to efficiently keep a representation of the target’s location in working memory (Noiret et al., 2018). In the context of patients with AD, it has been reported that they can predict a follow-up target, however, their anticipated saccades are more scattered around the target’s location (for a study on the PreS task and attentional control in AD see Mosimann et al., 2005; Noiret et al., 2018).

To conclude this section, the results of the presented studies identify SEM as liable biomarkers to early detect individuals at high risk of AD (Chehrehnegar et al., 2019, 2022; Wilcockson et al., 2019; Koçoğlu et al., 2021; Opwonya et al., 2022b). However, the available scientific literature is inconclusive about whether SEM tasks are useful to spot significant differences in gaze behavior between the MCI subgroups. While examining saccade metrics could be beneficial for guiding interventions aimed at treating older adults who are at a greater risk of developing MCI, more extensive studies with larger sample sizes are needed to confirm the clinical significance of SEM impairments in MCI (Koçoğlu et al., 2021). Similarly, longitudinal investigations are essential to (i) understand age-related cognitive changes and (ii) draw more definitive conclusions about the early detection of the transition from normal/healthy aging to MCI. Concurrently, by citing an interesting statement from the work of Everling and Fisher, one would like to assess whether it is essential to exclusively focus on saccadic tests: “*Despite a high sensitivity of the antisaccade task, its specificity for a disease or the location of the involved brain structure may be low* (…) (Everling and Fischer, 1998).” Therefore, besides SEM tasks, are there any other paradigms that are more suitable for differentiating between cognitively unimpaired and MCI populations? With this question in mind, the reader is invited to the next section of this review, dedicated to cognitively informative paradigms that may be of use in the future design of cognitive assessment tests.

### 6.2. Cognitively informative paradigms indicate eye movement impairments in MCI

As elucidated in the previous section, performing antisaccade tasks requires subjects to execute a goal-directed saccade in the opposite direction while suppressing the reflexive gaze towards the suddenly appearing stimuli. The antisaccade task has been considered a sensitive protocol to investigate inhibitory control and draw a line between HC and clinical populations, including individuals with AD and those suffering from MCI (Chehrehnegar et al., 2022). At the same time, it is hampered by low specificity. Abnormal gaze parameters such as an increased error rate have also been reported in the context of other disorders (Si et al., 2022). In the context of schizophrenia research, for example, the antisaccade task generates the most frequently observed volitional saccade abnormality (Levy et al., 2010).

Another limitation to consider is that antisaccadic eye movements have been reported as unnatural (Godijn and Kramer, 2007) and “*artificial by nature*” (Readman et al., 2021). To investigate how clinical populations approach daily life tasks, new research questions should require examination of paradigms that provide context-related exploratory eye movements in addition to the quantification of fixations and saccades (Readman et al., 2021; Wolf and Ueda, 2021; Wolf et al., 2021a). Also, the application of ecologically valid studies resembling real-life situations is surprisingly inadequate; hence, extensive investigations in lab-based and ecologically valid settings need to be conducted and reported in equally accessible publications.

Although the effectiveness of using eye-tracking technology to recognize individuals with MCI appears promising, in the past 5 years few research groups have implemented cognitively informative tasks. The following section is dedicated to studies that follow cognitively informative paradigms in order to differentiate between adults with MCI, AD, and HC, where (i) eye-movements represent an index for memory (for example, using the Visual Paired-Comparison task or Visuospatial Memory Eye-Tracking task), or visual attention and processing speed (King Devick test), and (ii) participants are challenged with a real-life situation (face recognition).

#### 6.2.1. Visual paired-comparison task

The human ability to identify, process, and ascribe greater attentional resources (attention bias) to novel stimuli is essential for exploring new opportunities and consequently adapting to changing environments (Eizenman et al., 2019). Therefore, the Visual Paired-Comparison (VPC) task offers the opportunity to provide complementary support to traditional composites for detecting early cognitive changes. In essence, the VPC task is an eye-tracking-based paradigm of particular interest due to its scientifically established method for detecting memory dysfunction in humans from infancy through adulthood (Pascalis et al., 1998; Manns et al., 2000; Crutcher et al., 2009; Zola et al., 2013). Furthermore, it has been shown that the VPC task reliably detects early signs of cognitive decline in older adults (Bott et al., 2017; Haque et al., 2019). In essence, a 30-min task quantifies how the participant splits attention between familiar and novel visual stimuli, with a familiarization phase preceding a testing phase.

In a study performed by Bott et al. (2018), subjects were presented with pairs of identical visual stimuli for 5 s (familiarization phase). Moreover, to assess immediate as well as delayed recognition memory, the test phase followed a delay of either 2 s or 2 min. During the testing phase, viewers were presented with additional pairs of visual stimuli, including one from the familiarization phase (familiar image) and one novel stimulus. Novelty preference (NP) defined the percentage of time the viewer spent looking at an unknown image compared with the image from the familiarization phase (thus, the ratio of time produces the NP score). A higher NP score represents a better declarative memory function, whereas a lower score indicates impaired function (Fantz, 1964; Fagan, 1970; Crutcher et al., 2009; Bott et al., 2018).

Individuals with MCI or AD have impaired declarative memory for previously viewed images and tend to spend an equal amount of time gazing at both novel and previously viewed (familiar) images. Conversely, individuals with normal cognitive function spend more time viewing novel images (photos not previously shown). Subsequently, one can assume that healthy older adults should not have notably lower scores on VPC tasks than younger individuals, as recognition memory remains stable with healthy cognitive aging (Danckert and Craik, 2013). On the other hand, individuals with MCI, AD, or even those who may have preclinical changes in cognition would be expected to score lower than unimpaired individuals (Bott et al., 2017, 2018; Gills et al., 2019).

Notably, performance on a 30-min VPC task demonstrated convergent validity between the eye-tracking test and cognitive composites that serve as preclinical AD indices, such as the Preclinical Alzheimer’s Cognitive Composite and NIH Toolbox for the Assessment of Neurological Behavior and Function Cognition Battery (NIHTB-CB). Exploring the influence of the used eye-tracker on task performance has been also underlined as a necessity, since it may impact the future application strategy (Bott et al., 2018). Indeed, the VPC test has been used in combination with commercial eye-trackers, which are capable of split-second monitoring of one’s gaze behavior, capturing an abundance of gaze metrics. However, it is essential to mention that high-quality equipment may be expensive and/or only available in research facilities, limiting the scalability of the clinical assessment. Therefore, Bott and colleagues underlined that an alternative and validated eye-tracking system needs to be proposed for feasible and widespread use.

A number of previous studies focused primarily on data obtained from commercial eye trackers. Notably, the investigation by Bott and colleagues presents modest-to-moderate correlations between VPC task performance using device-embedded cameras and scores on gold-standard cognitive composites. Device-embedded cameras offer a reliable and valid way to accurately assess VPC performance. Furthermore, since the strength of these relationships does not differ between types of camera devices, several researcher groups postulate that the ubiquity of cameras on most standard smart devices represents a scalable technique that is highly suitable for collecting population-level data (Bott et al., 2017, 2018; Gills et al., 2019). Correspondingly, with the growing number of smartphone and internet users (recent estimates indicate that there are over 5.44 billion smartphone users worldwide, equating to 68% of the world’s total population), positive developments pave the way toward improved healthcare in developing countries (Vashist et al., 2014). Scientists performing longitudinal studies on the early detection of MCI may consider cost-effective, remote eye-tracking options that empower personalized healthcare (Valliappan et al., 2020). Yet, above all, the next-generation digital diagnostic assessments must be thoroughly evaluated to guarantee their ethical, responsible, and professional use (Ahmed et al., 2015; Kasper et al., 2020; Kröger et al., 2020). While the enormous potential of nascent technologies should be acknowledged, an omnipresent use of eye-trackers will raise privacy concerns not only because gaze data may be collected and shared in non-transparent ways, but also because such data can contain a wealth of sensitive information about the viewer (for potential inferences that can be drawn from eye-tracking data refer to Kröger et al., 2020).

#### 6.2.2. The brief 5-min VPC test

Before proceeding to the detailed concept of the brief VPC test, it is worth mentioning that the VPC 30 falls into the category of passive paradigms, which means that participants complete the test without explicit instructions on where they are supposed to look. Accordingly, the test’s integrity depends on the user not knowing what the test is measuring. Therefore, it has been speculated that utilizing a shorter paradigm, in which participants are given specific instructions beforehand, would improve the user experience and increase the scalability of the assessment (Gills et al., 2019). A shorter and more active version of the VPC test has thus been established.

In the brief 5-min VPC test, before the testing phase begins, participants are instructed to focus their gaze on the new image (novel stimulus). While this quick test has been previously validated to evaluate declarative memory function among healthy individuals, it remains unknown whether this test accurately discriminates between cognitively healthy and cognitively impaired older adults. Therefore, Gills and colleagues aimed to determine the ability of the eye-movement metrics obtained from the 5-min VPC test (*via* a factory-installed web camera) in distinguishing between cognitively normal and cognitively impaired adults (Gills et al., 2021). Their results demonstrated the brief VPC task to be a helpful screening tool for cognitive impairment that can be used to accurately assess memory function. Besides noteworthy correlations with the MoCA, the brief VPC task is characterized by significant correlations with individual NIHTB-CB tasks measuring inhibitory control and attention, processing speed, and visual episodic memory. Moreover, the researchers could successfully discriminate between cognitively impaired and cognitively normal individuals irrespective of age. Finally, the brief version gives a premise of high test–retest reliability (Gills et al., 2021).

Another independent study, which aimed to assess differences in gaze behavior between healthy elderly individuals and patients with MCI, has been conducted by Nie et al. (2020). In line with previous investigations, the research group assessed the NP score in the VPC eye-tracking task and concluded that this parameter is a simple and non-invasive diagnostic biomarker of MCI. The NP score accurately distinguishes patients with MCI from cognitively normal subjects. Notably, when assessing the NP after either a 2-s or 2-min delay, AUC analysis showed that an NP score of 0.605 in the 2-min-delay condition effectively differentiated participants with MCI from HCs. Echoing previous findings (Crutcher et al., 2009), Nie and colleagues reported that novelty preference differs significantly between HCs and participants with MCI when the delay period is 2 min but not 2 s. Moreover, this difference remained significant at two-week follow-up. In conclusion, the method achieved a specificity of 72% and sensitivity of 53% (Nie et al., 2020). Furthermore, nine participants with poor novelty preference scores (whose novelty preference score fell below the 0.605 cut-off point at the initial testing) showed significant decline in cognition during 1-year follow-up (Nie et al., 2020).

**Figure 4:**
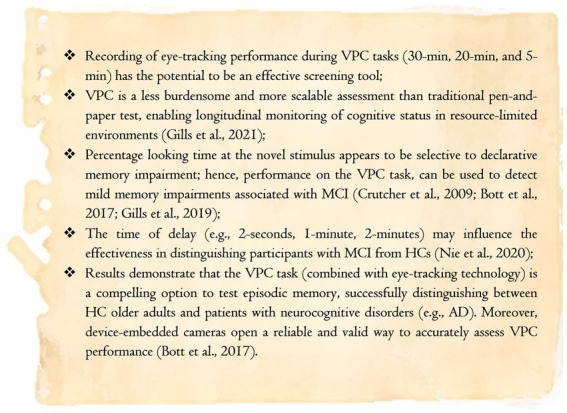
Take-home messages for the section dedicated to the Visual Paired-Comparison test (own elaboration based on reports from Bott et al., 2017; Gills et al., 2019, 2021; Nie et al., 2020).

Due to the lack of objective indicators and boundaries between MCI and cognitively healthy elderly individuals, distinguishing between these groups can be more challenging than diagnosing dementia (Seligman and Giovannetti, 2015; Nie et al., 2020). Nevertheless, with cognitive examinations increasing in popularity (Gills et al., 2021), VPC paradigms unfold valuable screening tools for assessing and tracking cognitive status over time. In addition, the short VPC task is clinically valuable despite not being widely available. Combined with near-infrared eye-tracking apparatus or device-embedded cameras, VPC tasks may identify seemingly cognitively healthy subjects in whom MCI is underdiagnosed. The brief VPC has been reported to be well-tolerated by participants due to the shorter testing times (the test requires only 5–10 min to complete, including calibration). To conclude this section, investigations in the memory recognition domain open new perspectives to study cognitive disturbances in clinical populations (refer to the take-home notes in Figure 4). Despite the fact that further longitudinal clinical studies are needed, novelty preference scores have surfaced as an easily accessible physiological marker for MCI (Crutcher et al., 2009; Bott et al., 2017; Gills et al., 2019).

#### 6.2.3. Visuospatial memory eye-tracking task: a screening tool for cognitive impairment and AD status

Since pathological changes in AD develop years before the onset of clinical symptoms, the preclinical AD period has generated considerable interest in detecting subtle memory impairments (Dubois et al., 2016; Parnetti et al., 2019). Therefore, Haque and colleagues sought to develop an easily administered, enjoyable, and sensitive paradigm for passively assessing mild memory deficits at an early stage of the disease course. In particular, the authors followed the suggestion that visuospatial memory paradigms are sensitive indicators of hippocampal-dependent memory function decline (Small et al., 2000; Yassa et al., 2011; Reagh et al., 2016; Hampstead et al., 2018) and, therefore, may serve as an early indicator of memory impairment in AD.

Previously, paradigms that investigated eye movements as an index of memory retrieval requested participants to view a set of images (encoding phase) and their manipulated (or not) versions (objects added, removed, or moved; Ryan et al., 2000; Smith and Squire, 2008). Notably, regarding the repeated images, it has been reported that participants spend more time viewing the manipulated regions compared to the unchanged regions. These results suggest that eye movements rather than explicit memory judgments are suitable for assessing visuospatial memory and evaluating its performance among healthy controls and memory-impaired subjects. Furthermore, more recent studies support the use of eye movements as an indicator of memory dysfunction (Crutcher et al., 2009; Hannula et al., 2012; Zola et al., 2013; Pathman and Ghetti, 2015; Pavisic et al., 2021).

**Figure 5:**
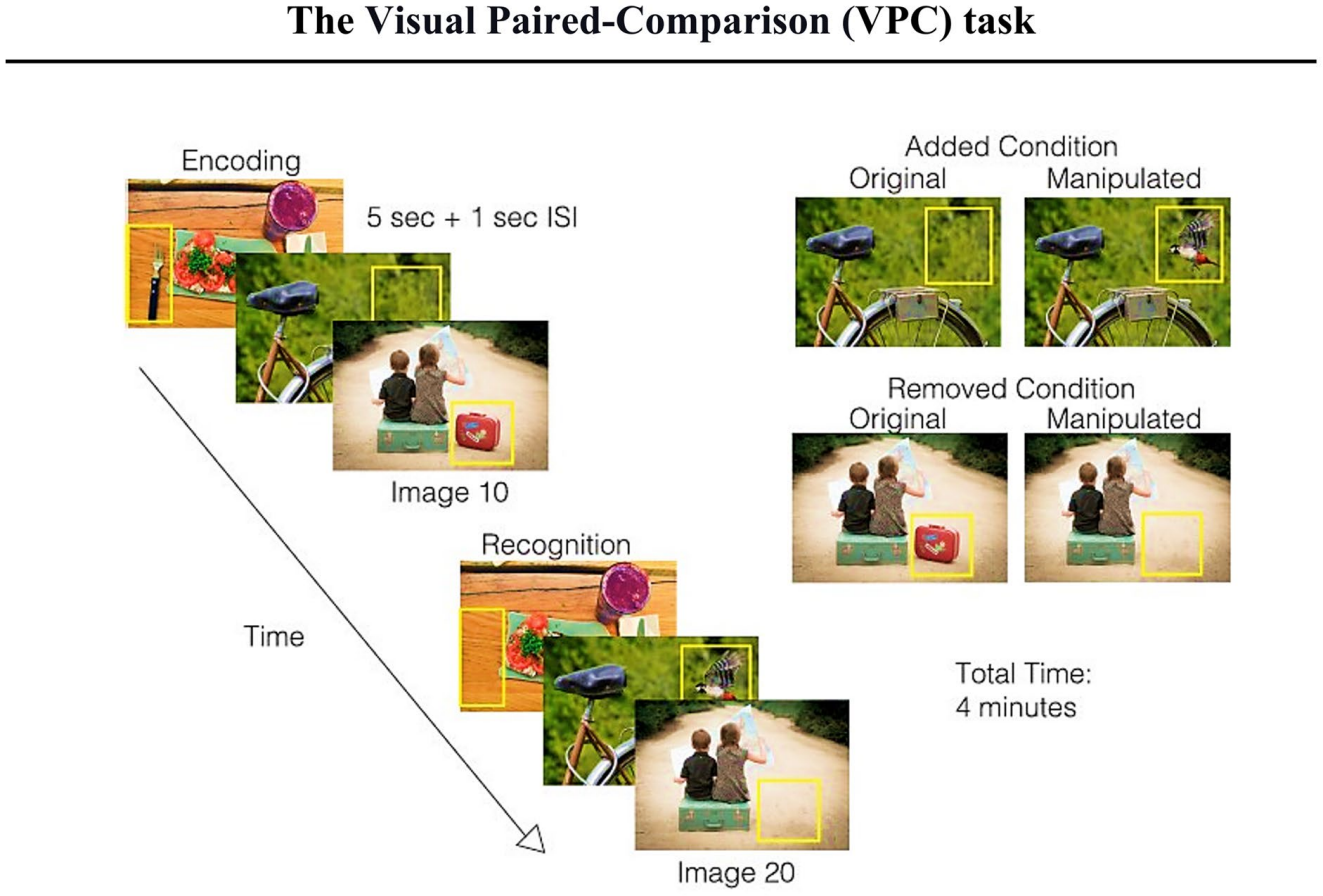
Schematic of the visuospatial memory eye-tracking task with a brief explanation (figure of the paradigm taken from Haque et al., 2019). Participants are asked to view a set of images for 5 s (with a 1 s interstimulus interval during the encoding phase). During the recognition phase, participants view the same set of realistic images with either one item removed (removed condition) or one item added (added condition). The manipulated regions (indicated by the yellow box just for an explanatory reason) are used to quantify memory performance. The final test consists of the presentation of two sets of 10 original and manipulated pairs (seven with removed condition and three with added condition) with a delay of 1 min between the original and manipulated presentations. The entire task takes 4 min.

Hence, building on these scientific contributions, Haque and colleagues developed the Visuospatial Memory Eye-Tracking Task (VisMET), during which participants perform a memory paradigm that relies solely on participant’s eye movements (Figure 5). During the encoding phase, participants are instructed to “enjoy” viewing a set of naturalistic images. It is crucial to note that VisMET requires memory for a complex set of associations between objects and locations and is assessed passively using eye movements rather than requiring explicit memory judgments. Participants are not informed that they have been given a memory task. In the recognition phase, participants view a modified version of the same set of images with either an item removed (*removed condition*) from the photo or an item added to the image (*added condition*). Importantly, to minimize the impact of one’s eye movements from the central fixation cross of the calibration screen preceding each image, the authors reported modifications being applied to noncentral locations only.

The amount of time viewing the manipulated regions of interest, compared to unchanged regions of the images, can be used to measure memory of either a previously viewed object and location (removed condition) or a new object and location (added condition; Figure 5). Moreover, Haque and colleagues speculated whether obtained performance score could be used as a screening tool for identifying MCI and AD states. Therefore, the 4-min paradigm has been primarily administered to 296 control and memory-impaired participants (MCI or AD) with the aim to compare visuospatial memory performance in healthy aging and at different stages of AD. When training the models to predict cognitive impairment (MoCA ≤23), the researchers found that VisMET performance was able to achieve an AUC of 0.85 compared to an AUC of 0.71 and 0.56 when using age and education, respectively. This model was able to achieve a sensitivity of 0.83 and specificity of 0.74, using a cutoff probability of 0.64. To further evaluate VisMET, researchers aimed to determine the sensitivity of VisMET performance in predicting disease status, where the output of the model was the diagnostic classification of healthy control, MCI, or AD. By training a logistic regression classifier with the same three features as before, memory performance predicted MCI/AD status with an AUC of 0.85 compared to 0.73 and 0.58 when using age and education alone. Notably, after taking into account all of the features, the achieved sensitivity and specificity were 0.85 and 0.75 respectively, with a cut-off probability of 0.63 (Haque et al., 2019).

In conclusion, Haque and colleagues raised a number of important results, including that memory performance on the VisMET task is (1) different between healthy and MCI/AD participants, and (2) dependent on the difficulty in interpretation of the original and manipulated images. In relation to the latter aspect, since difficulty can be manipulated, it may allow VisMET to be sensitive across a broad range of memory abilities. Furthermore, VisMET performance has been reported to be age-dependent. The group of people aged 50–59 years performed better on the memory task than those aged 60–69 and 70+ years. Moreover, the percentage of critical regions viewed by the 50–59 years age group differed statistically when compared to the 60–69 years (*p* < 0.001, unpaired t-test) and the 70+ years age groups (*p* < 0.01, unpaired t-test). Concurrently, there was no difference in performance between the age groups 60–69 and 70+ years. Finally, a multivariate model of memory performance on the task predicted cognitive impairment and AD status with high sensitivity and identified a subpopulation of healthy controls with relatively weak performance on the task.

Following these promising results, to enable efficient and widespread administration of the VisMET task Haque and colleagues developed a mobile version of the memory paradigm. VisMET has been delivered on iPad devices to assess cognitive status in a population of 250 individuals (Haque et al., 2021). The authors used a transfer learning approach to train a deep neural network to track participants’ gaze behavior. In conclusion, mild-to-severe cognitive impairment was identifiable with a test accuracy of 70%; furthermore, by enforcing a minimal calibration error of 2 cm, an accuracy of 76% was achieved. It is important to mention that this result has been reported to be equivalent to the accuracy obtained using commercial eye-tracking hardware. Overall, these data demonstrate a mobile VisMET version that can estimate the presence of cognitive impairment (Haque et al., 2021). With the widespread use of smart devices as a non-pharmacological intervention (Astell et al., 2019), future advancements in technology combined with eye-tracking may offer new opportunities for detecting the onset of an abnormal aging process (Bott et al., 2018; Boyd et al., 2021) as well as visual impairments linked to other disorders (Wolf and Ueda, 2021; Wolf et al., 2021a) on a worldwide level.

#### 6.2.4. King Devick test

Due to cognitive deficits in information processing, memory, and visual learning, a commonly used instrument to measure information processing speed is the King Devick (KD) test, which has been reported to be sensitive in detecting performance change in clinical populations. It comprises a simple visual-verbal task that requires precise saccades and intersaccadic fixations. Previous research has shown the KD test’s performance to be correlated with the Symbol Digit Modalities Test (SDMT) as well as MoCA scores.

**Figure 6:**
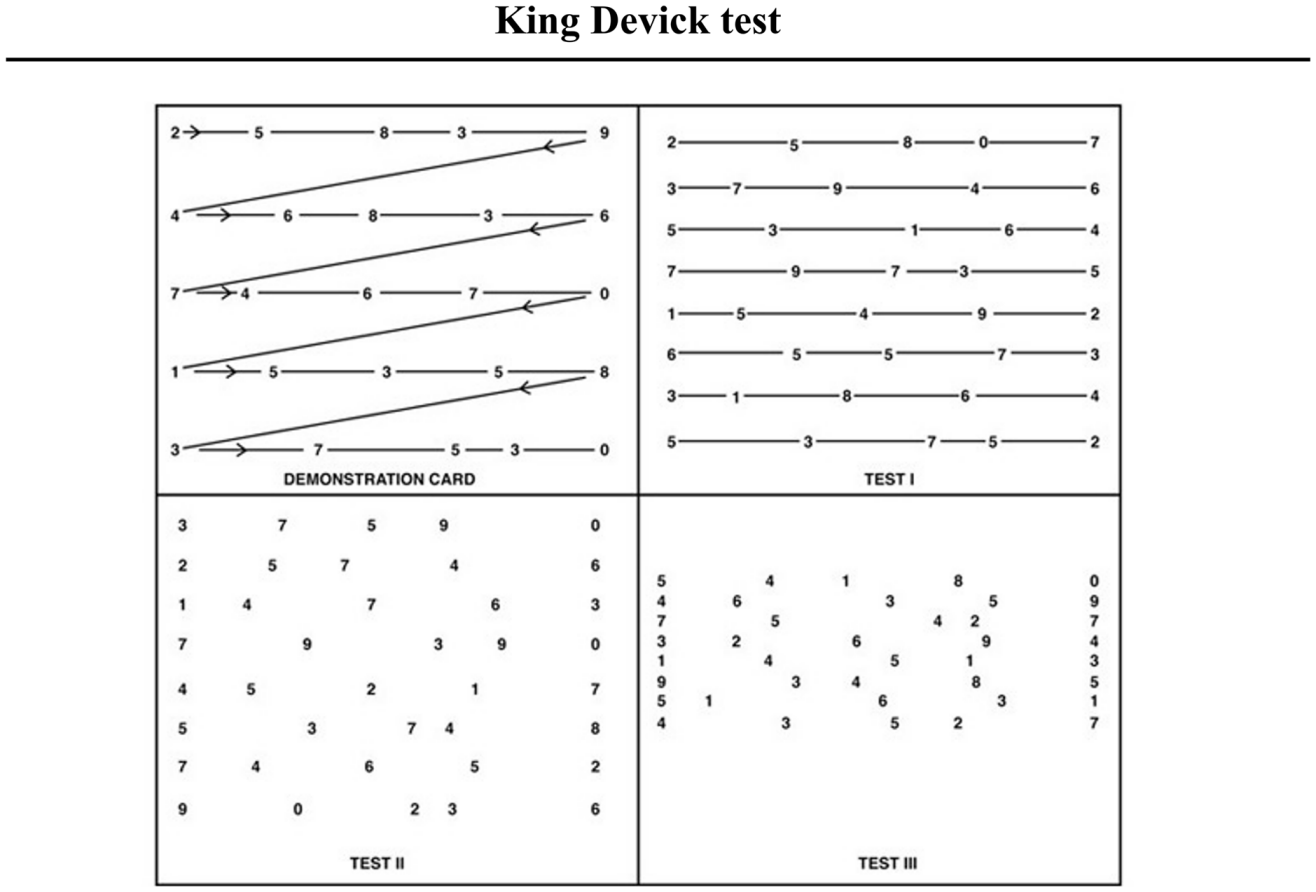
Schematic view of the King Devick test with a brief explanation (figure obtained from Leong et al., 2015). Each test card displays 40 digits in five rows, with the spacing between each number varying between rows and across rows. Notably, the visual demands of the test cards increase as the test progresses. The first test card has straight lines connecting the numbers that aid visual scanning. In the second test card, the lines connecting the numbers are missing. The final test card is made up of numbers with no connecting lines and with the spacing between the rows truncated.

In short, the KD test is a 1–2-min, rapid number naming test, often used to assist cognitive impairment in multiple sclerosis or after concussion (Galetta et al., 2015; Gold et al., 2021). Notably, it also has clinical utility in other conditions such as Parkinson’s disease and AD. This visual scanning test requires participants to read numbers out loud as quickly as possible. Commonly, there is one demonstration card at the beginning, followed by 3 test cards that become progressively more difficult due to changes in spacing and vertical crowding of the numbers (Figure 6). Each card increases visual demands and allows interference from other rows as the participant reads across the page. Scores are generated based on the total time taken to complete the test. A higher score indicates worse performance where aged-normed T-Scores ≤40 are classified as borderline or impaired.

As previously mentioned, impaired eye movements may be an early indicator of AD (Molitor et al., 2015; Kahana Levy et al., 2018; Hannonen et al., 2022; Opwonya et al., 2022b) with saccadic eye movement impairments being one of the most commonly documented forms of oculomotor dysfunction among patients with AD (Fernández et al., 2013; Chang et al., 2014; Galetta et al., 2017). Additional studies have also demonstrated that patients with aMCI exhibit abnormal saccades resembling mild AD (Peltsch et al., 2014; Wilcockson et al., 2019). These findings raise the possibility that testing goal-directed eye movements may have strong utility in the detection of cognitive impairment (Readman et al., 2021). Since the KD test requires participants to perform precise, horizontal eye movements coupled with a rapid number naming task, it’s score may provide an early indicator of an overall cognitive impairment, where impaired individuals are expected to have a greater number of errors and take more time to complete the number naming task (Lin et al., 2014). In short, the KD test score is the total time required (in seconds) to complete three test cards, where higher scores reflect worse performance (Lin et al., 2014; Galetta et al., 2017; Gold et al., 2021).

The first research group to test the utility of the KD in AD was Galetta et al. (2017). The sample included 135 HCs and 71 cognitively impaired patients (MCI = 39, AD = 32), AUCs generated from logistic regression models revealed that the KD test can distinguish controls from cognitively impaired subjects (MCI AUC = 0.71; AD AUC = 0.74). KD time scores between 48–52 s were associated with high sensitivity (>90.0%) and negative predictive values (>85.0%) for each diagnostic group. The research group concluded that the KD test is a simple and effective screening tool to detect cognitive impairment associated with AD in an efficient time frame (Galetta et al., 2017). Moreover, worse performance on the KD test may capture distinct pathological changes related to AD that affect saccadic oculomotor function. Nevertheless, these preliminary results await further validation through empirical testing.

Recently, the KD test has been used to examine whether obtained gaze metrics (saccadic duration and amplitude) can differentiate cognitively healthy control groups from subjects with minor changes on cognitive tests or those diagnosed with mild AD (Hannonen et al., 2022). Hannonen and colleagues recruited 57 non-demented participants and 21 patients with mild AD (Hannonen et al., 2022). All subjects underwent neurological examination, including the Consortium to Establish a Registry for Alzheimer’s Disease neuropsychological test battery (CERAD-NB) and a Clinical Dementia Rating interview. Furthermore, the non-demented participants were divided into two groups, namely control (normal CERAD subtests, mean MMSE = 28) and objective MCI (decline in at least one CERAD memory score, mean MMSE = 27). The research group found significant differences between the three groups (control, objective MCI, and AD) in regard to the mean saccade amplitude (3.58, 3.33, and 3.21 ms, respectively, *p* < 0.03) and duration (27.1, 25.3, and 24.8 ms, respectively, *p* < 0.05). Furthermore, the KD error scores of AD patients differed significantly (*p* < 0.01) from the other groups (Hannonen et al., 2022).

**Figure 7:**
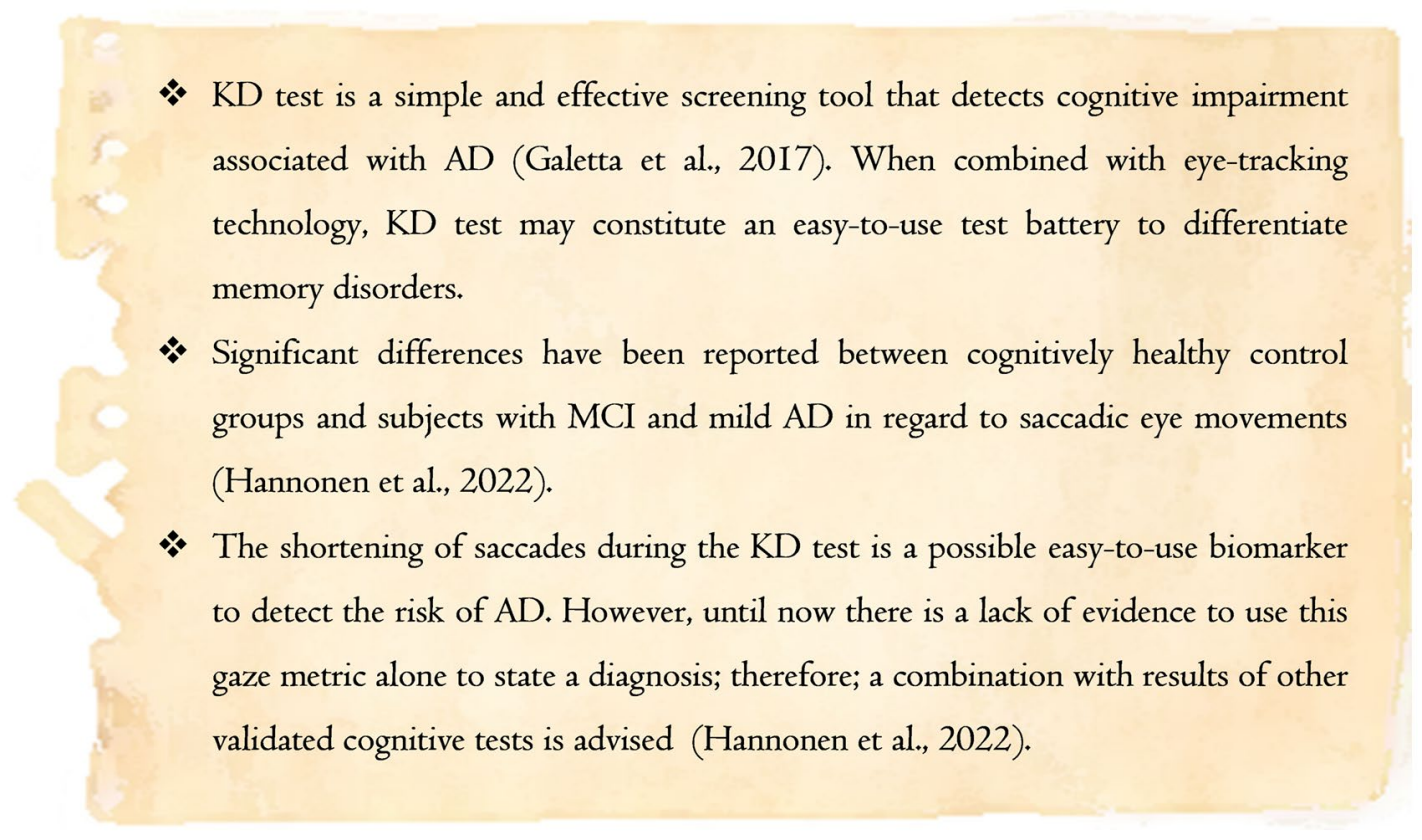
Take-home-messages for the section dedicated to the King Devick test (own elaboration based on scientific works by Galetta et al., 2017; Hannonen et al., 2022).

Overall, the results from KD testing provided the scientific community with some practical insights regarding future practices related to eye-tracking technologies (refer to the take-home notes in Figure 7; Galetta et al., 2017; Hannonen et al., 2022). The previously reported notion of eye-tracking technology adding value to the screening process in MCI has been unanimously supported. Moreover, the convenience of portable eye-tracking devices for future use in primary health care memory clinics has been highlighted (Hannonen et al., 2022). However, considering the accuracy of the KD test as a screening tool and large in-group variances among participants, neither saccadic duration nor saccadic amplitude alone can faultlessly classify cognitively unimpaired individuals. For now, it is advised to use these two parameters in combination with other screening tools (Hannonen et al., 2022).

#### 6.2.5. Visual impairments in face processing tasks

Eye-tracking represents a category of interdisciplinary research that successfully combines with various tasks. It can also evaluate human gaze behavior in association with numerous stimuli categories, such as geometrical figures, illusions, and pictures of computerized human faces (Simion and Shimojo, 2006; Prats et al., 2010; Borji et al., 2013; Gidlöf et al., 2013; Spinks and Mortimer, 2015; Vriens et al., 2020; Wolf and Ueda, 2021). Yet, the use of abstract stimuli may reduce the ecological validity of a neuropsychological study, defined by Sbordone and Long in 1996 as “*the functional and predictive relationship between the patient’s performance on a set of neuropsychological tests and the patient’s behavior in a variety of real-world settings (e.g., at home, work, school, and community)*” (p. 16; Sbordone, 1996; Diaz-Orueta et al., 2022). Hence, few research groups opt to use realistic stimuli to investigate visual processing among adults with MCI.

For example, Kawagoe and colleagues requested study participants (aMCI and HCs) to judge whether two images (faces or houses) were the same or different (*perception study*). In the follow-up task, the participants were asked to indicate which of the two images, if any, had been presented previously (*short-term memory study*). The results showed that, when judging whether the images were the same or different, HCs spent more time visually inspecting the eye and nose. Notably, this effect was not observed among older adults with aMCI, who looked longer at the mouth area. When judging whether an image had been previously presented, the observed fixation pattern of facial landmarks did not differ between groups (HC and aMCI), yet patients with aMCI showed a decline in memory for faces but not for houses (Kawagoe et al., 2017).

In 2018, McCade and colleagues introduced a novel eye-tracking paradigm to investigate if deficits in emotion recognition are evident among individuals with MCI. For that reason, the research group used naturalistic stimuli in the form of emotional faces (NimStem Set of Facial Expressions) to introduce recruited participants (18 HC, 18 patients with naMCI, and 14 patients with aMCI) to a free visual search paradigm. Although older adults with aMCI were less accurate on emotion recognition than HC and naMCI, no significant difference in mean fixation durations on eye, mouth, and peripheral facial regions was reported. Gaze behavior analysis revealed all participants showing a preference for the eye region. Interestingly, while visually exploring disgusted and angry faces, fixation time on the eye region was significantly shorter for all groups (McCade et al., 2018). In comparison to HC and naMCI, participants with aMCI were less accurate in recognizing the emotion of all categories of presented facial stimuli (McCade et al., 2018). The result of poorer performance in emotion recognition among individuals with aMCI has been replicated in another independent study introducing a computer-based emotion recognition test for older adults (HC = 69; AD = 84; and aMCI = 59), where the processing speed score from the Affect-GRADIOR test has been reported to slightly improve the predictive power of the MMSE (García-Casal et al., 2019; for an excellent review on emotion recognition and processing in MCI patients refer to Morellini et al., 2022).

In summary, forming conclusions regarding the efficacy of face processing paradigms as an early diagnostic tool is limited due to the shortage and high variability of currently available scientific literature (Readman et al., 2021). Nevertheless, the inclusion of naturalistic stimuli and tasks that mimic instrumental activities of daily living such as face recognition or social conversation is of great interest (Kim et al., 2019; Miyake et al., 2020; Sayma et al., 2020; Oliveira et al., 2021; Otake-Matsuura et al., 2021). By transforming the available protocols into real life scenarios, ecologically valid results can be generated (Tarnanas et al., 2013; Sonkusare et al., 2019). Moreover, the adaptation of naturalistic stimuli in neuroscience continues to promise exciting new applications integrating ecologically valid paradigms with VR protocols (Kim et al., 2019; Sonkusare et al., 2019; Sayma et al., 2020; Oliveira et al., 2021; Readman et al., 2021; Lee et al., 2022).

### 6.3. Combination of eye-movement tests

To commence this section, the authors would like to quote a pertinent observation made by Arolt and colleagues that, although made in relation to schizophrenia research, is highly relevant to the investigation of cognitive deficits among other clinical entities. *“It has to be kept in mind that each of the mentioned deficits is nonspecific, but can occur in a variety of brain diseases. With regard to the literature on eye movement dysfunction in schizophrenia, it is obvious that not by one single task, but possibly by their combination, eye movements might serve as a biologically based diagnostic tool, in addition to psychopathology”* (Arolt et al., 1998). In the mentioned citation, Arolt strongly emphasizes that a single task may not serve as a reliable diagnostic tool. A similar conclusion shapes the direction of recent projects related to MCI and AD research, where a combination of gaze metrics obtained from multiple tasks may increase the classification accuracy to distinguish patients from HC (Oyama et al., 2019), as well as characterize MCI subtypes.

Since it has consistently been demonstrated that eye movements differ between individuals with AD and healthy controls, and that performance might be associated with attentional factors, two independent research groups employed a series of paradigms to match the following requirements: (i) reproductivity, (ii) inclusion of scientifically recognized tasks, and (iii) implementation of attention-demanding components (including working memory, attention and calculation tasks, and visual working memory tasks) that are suspected to help differentiate the groups (Oyama et al., 2019; Tadokoro et al., 2021).

#### 6.3.1. Novel methods for the rapid assessment of cognitive impairment

To assess cognitive function supported by eye-tracking technology, Oyama and colleagues developed a novel cognitive assessment tool. The cognitive function of HC (*n* = 27), MCI participants (*n* = 26), and patients with dementia (*n* = 27) were assessed (mean MMSE scores were 28.7, 25.7, and 16.0, respectively). Moreover, a subset of participants underwent cognitive assessments such as the ADAS-Cog, Frontal Assessment Battery (FAB), and Clinical Dementia Rating where patients with MCI and dementia performed significantly worse than healthy older adults. According to the methodology, all participants were asked to view a series of short movies and pictures displayed on a screen. Since the total assessment time was approximately 3 min, the screening tool has been reported as practical and brief (Oyama et al., 2019).

**Figure 8:**
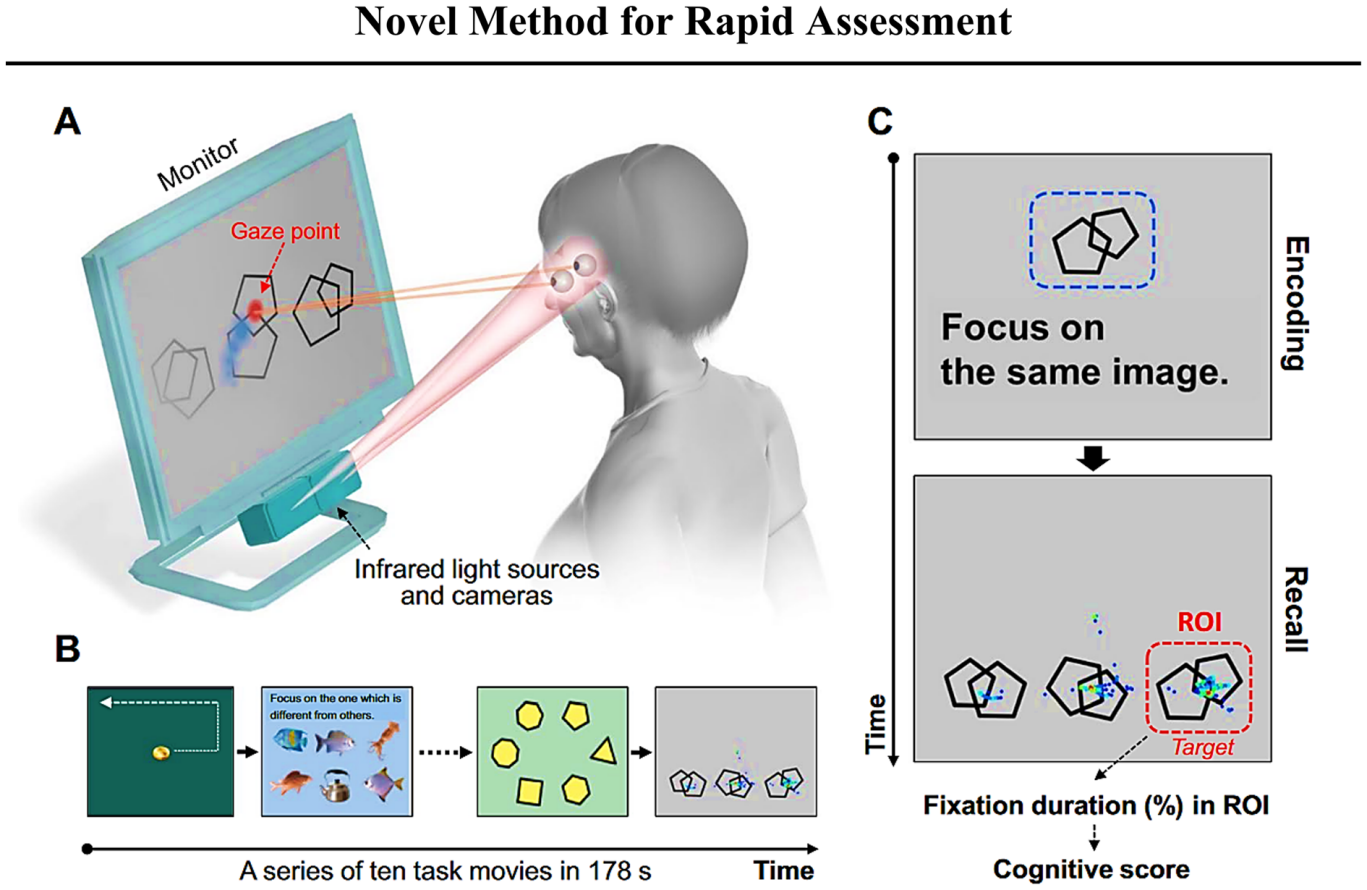
Schematic view of the Rapid Assessment of Cognitive Impairment Test, obtained with permission from Oyama et al. (2019). **(A)** Rapid cognitive assessment using an eye-tracking technology and ten task movies. **(B)** The subject views a series of tasks and pictures (for a total of 178 s), which assess smooth pursuit eye movement, deductive reasoning, visuospatial function, and working memory. **(C)** An example (Task 4) of the visual working memory task (pattern matching). The participant is asked to look at target image (here: a combination of a circle and a triangle) for 10 s (encoding) in order to correctly recall the object later on. The fixation duration within the region of interest (ROI) of the target object is used to calculate the cognitive score. For full details of the procedure kindly refer to the supplementary information in Oyama et al. (2019).

A series of 10 short movies and pictures, each designed to assess specific neurological domains, were used in this rapid assessment test. In each task the target image (correct answer) and non-target images (distractors) were presented on a monitor. The subjects were instructed to identify and focus their gaze on the correct answer (see Figure 8 for a schematic view of the paradigm). The idea behind it is simple and straightforward, a region of interest (ROI) was set on the correct answer (target image), and the percentage fixation duration on the ROI was used to calculate the cognitive score. Importantly, valid gaze detection data (not the total exposure duration) was used to determine the cognitive score, considering loss in data due to blinking or looking outside the monitor area. In result, the assessed cognitive scores showed a strong positive correlation with MMSE scores (*p* < 0.00001), and correlated well with scores from the ADAS-Cog, FAB, and Clinical Dementia Rating, showing an outstanding diagnostic performance in detecting patients with dementia (Oyama et al., 2019).

In 2021, Tadokoro and colleagues examined the utility of an eye-tracking test resembling that presented by Oyama et al. (2019). During each procedure, 10 tasks were displayed one-by-one for a total of 3 min on the computer monitor. Again, as in the pipeline presented by Oyama et al., 2019, each subject was required to look at the monitor while the eye-tracking device recorded their gaze points through infrared light cameras. Several ROIs were set, representing the locations of correct answers, incorrect answers, and the explanatory text for each task (specifically for tasks: #1-b, #3, #5, #6, #7, #9, and #10). Total score and subscale scores of delayed recall, working memory, judgement, and visuospatial function (range 0–100; higher means better) were automatically calculated (Tadokoro et al., 2021). In addition, Tadokoro and colleagues evaluated cognitive function of healthy controls (*n* = 52) *via* MMSE, alongside patients with MCI (*n* = 52) and AD (*n* = 70). It was reported that eye tracking scores declined significantly in individuals with MCI (*p* < 0.01 vs. HCs) and AD (*p* < 0.01 vs. HCs, *p* < 0.01 vs. MCI), and correlated well with the MMSE score (*p* < 0.05). Notably, the total score was an average of only four tasks as, according to the article, gaze metrics obtained in tasks #2 [moving coin video], #4 [free viewing of a static landscape photograph], and #8 [an animation of a falling water drop] were not used to calculate the total score.

AUC values were calculated from the ROC curve as an indicator of diagnostic value. In addition to the goal-directed tasks (to select a correct answer: #1-b, #3, #5, and #7), the moving coin task (#2) also showed a high AUC. These results align with previous reports on impaired smooth pursuit in AD. Notably, some of the goal-directed tasks (task numbers #6, #9, and #10) did not effectively distinguish between HCs, MCI, and AD; the authors pointed at the low difficulty level as a possible reason. Therefore, in order to keep the screening procedure as time restricted as possible, Tadokoro and colleagues suggested to omit ineffective tasks (#4, #6, #8, #9, and #10) while implementing their paradigm into future screening applications (Tadokoro et al., 2021).

**Figure 9:**
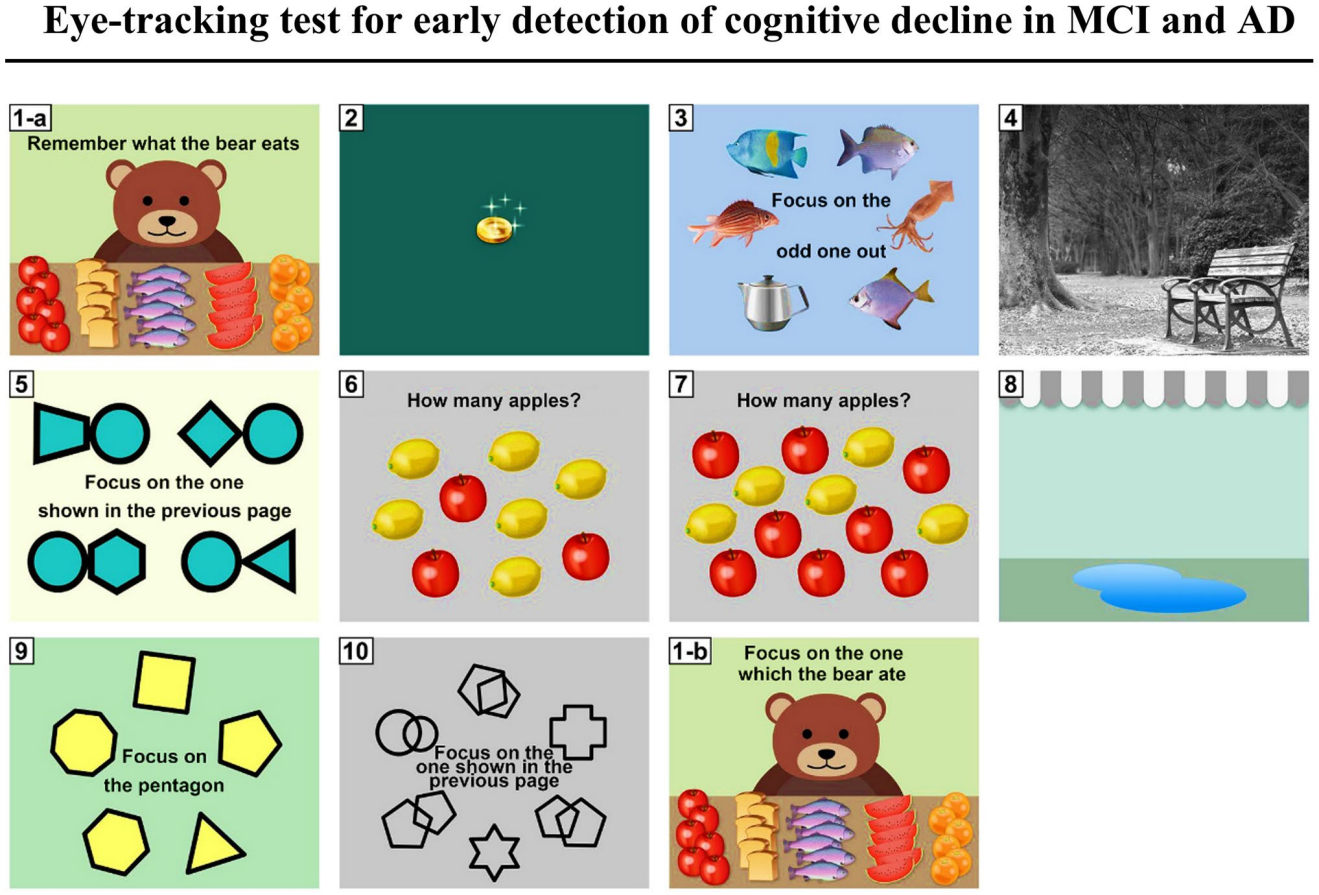
Eye tracking test for the early detection of cognitive decline in mild cognitive impairment and Alzheimer’s disease, obtained with permission from Tadokoro et al. (2021). Representative images of all 10 tasks with English instructions, which were initially given in Japanese. For full details of the instructions, see Tadokoro et al. (2021).

Interestingly, the landscape photograph task (landscape photograph displayed without instructions, see task #4 in Figure 9) has been reported to fail in exerting a good diagnostic power (Tadokoro et al., 2021); suggesting the possibility that the landscape scene was too simple and/or of low interest to the viewers. Indeed, previous scientific reports mentioned eye-movement impairments among AD patients while looking at naturalistic pictures. However, such observation refers primarily to the diminished curiosity aspect (Przybyszewski et al., 2023). Another point for emphasis is the specific protocol of the free-viewing task, which requires participants to freely view a given scene without explicit instructions (such as *a photograph of a bench in a park*). Such choice of procedure removed the requirement for any potential influences that could dictate where participants should direct their gaze (Tadokoro et al., 2021). Nonetheless, keeping the assignment simple and instruction-less makes it difficult to conclude whether a task is assessing a specific cognitive domain or whether participants’ abilities (or impairments) influence their performance on a task. Goal-directed free-viewing paradigms, on the other hand, have the potential to robustly identify cognitive impairment in preclinical stages (Manns et al., 2000; Dragan et al., 2017; Readman et al., 2021). Hence, although the presented studies (Oyama et al., 2019; Tadokoro et al., 2021) demonstrate practical eye-tracking tests for grading the cognitive state of older adults, their scientific conclusions clearly show a direction for further improvements (for example, modifying/replacing some blocks). Finally, in order to transform valuable observations related to atypical gaze patterns into applicable cognitive scoring tests, it is essential to carry out longitudinal studies in laboratory- and home-based settings, and report the results in equally accessible publications (Sbordone, 1996; Tarnanas et al., 2013; Sun et al., 2022).

## 7. Discussion

### 7.1. … on addressing eye-tracking-based screening

Until now, the exact mechanisms of how and why various forms of dementia develop remain unclear. Disappointing results of clinical trials for putative new treatments for AD combined with growing evidence of a decade-long preclinical stage of AD have led the scientific community to develop screening tools with high sensitivity and specificity as well as preventive countermeasures (Tarnanas et al., 2013; Galetta et al., 2017; Palsetia et al., 2018; Rutkowski et al., 2020; Thabtah et al., 2020; Lee et al., 2022). Undoubtedly, the accurate prediction of which older adults will progress to develop AD would mark a breakthrough by maintaining their independence (Langa and Burke, 2019). Since, in the context of AD, it is desirable to reach a diagnosis before the disease has progressed to involve massive neuronal loss in the brain, identification of the intermediate phase plays an important role in early intervention, prevention, and treatment (Ataollahi Eshkoor et al., 2015). Hence, it is imperative to develop user-friendly cognitive scoring tools that would aid clinicians to accurately identify and classify a neurodegenerative condition as early as possible (Marandi and Gazerani, 2019; Alber et al., 2020; Majeed et al., 2021; Klyucherev et al., 2022).

A growing body of evidence suggests that gaze metrics are useful in the screening of individuals at risk of diseases, including AD, Parkinson’s, Autism spectrum disorder, and nystagmus syndrome (Rosengren et al., 2020; Kong et al., 2022; Sun et al., 2022). Further investigations of specific eye movement biomarkers and neuropsychological criteria that precisely separate MCI subtypes (aMCI and naMCI) may assist in the forecasting of dementia progression. Along the same line, a better understanding of MCI subtypes could facilitate the development of targeted prevention strategies and offer a more effective approach for testing the efficacy of future therapeutic interventions (Busse et al., 2006; Csukly et al., 2016; Wright and O’Connor, 2018; Clark et al., 2019).

Future eye-tracking-based experiments may address the challenges and aim to expand the knowledge of differential diagnostics. While it remains speculative if gaze metrics will ever be used as a standalone diagnostic criterion (Clark et al., 2019), experimental paradigms that take into account one’s eye-movement behavior (Rodrigue et al., 2018; Clark et al., 2019; Wolf et al., 2021a) have appeared to shorten screening procedures and improve diagnostic accuracy (Lagun et al., 2011; Zola et al., 2013; Lauermann et al., 2017; Ehrlich et al., 2022). Yet thought-provoking is the fact that although visual and oculomotor problems are prevalent among older adults, and gaze recordings may support clustering various clinical problems, e*ye-tracking* technology is somehow excluded from routine screening investigations (Tsitsi et al., 2021) and remains in the academic dimension only (Wolf et al., 2021a). Furthermore, despite available reports on improved eye care going hand-in-hand with an improved dementia prevention strategy, information about visual impairments is not used to shape public health policy nor research priorities of dementia risk factors (Ehrlich et al., 2022; The Lancet Regional Health – Europe, 2022). An increase in awareness about the diagnostic value of one’s gaze and knowledge in interpretations of gaze behavior abnormalities is crucial (Cañigueral et al., 2019; Holmqvist et al., 2022); especially, that routine eye-movement-based cognitive assessments, which provide a quantitative and objective method to aid diagnoses in older adults, are technically feasible (Galetta et al., 2017; Oyama et al., 2019; Dickens and Ramaesh, 2020; Tadokoro et al., 2021).

Monitoring potential changes in the performance of eye-movement tests may facilitate the identification of older adults who are at risk of developing AD, becoming a valuable tool for primary health care clinics (Molitor et al., 2015; Holden et al., 2018; Wilcockson et al., 2019; Pereira et al., 2020; Lehtola et al., 2022). It has been reported that such recordings could be performed in eye care clinics equipped with cost-effective eye trackers (Rosen, 2004; Dickens and Ramaesh, 2020) or at home *via* devices with built-in cameras such as smartphones or tablets. Following the dramatic increase in the use of consumer electronics by aging adults, digital approaches that leverage the capacities of mobile devices and internet connectivity represent a promising direction for detecting MCI in non-clinical environments. To support this concept, mobile versions of several tests have been reported to demonstrate a high capability of estimating the presence of cognitive impairment (Bott et al., 2017; Sabbagh et al., 2020a; Haque et al., 2021). Hence, it is becoming increasingly possible to detect visual impairments associated with neurodegenerative disorders on a global level. Paving the way for computer-based diagnosis and prognosis, eye tracking facilitates the automation of medical decision support. Such a multimodal approach would increase the range of screening possibilities for older adults, although proposed assays need to be adequately validated and linked to healthcare systems with equity.

The authors of this review echo the conclusions of previous works that the static image of the eye can provide the scientific community with information regarding physiological changes in the brain. However, the pathological changes in the retina are difficult to associate with a singular disease. On a dynamic scale, however, eye movements can provide valuable hints to understand one’s cognitive functioning and narrow the possible diagnostic options. Unveiling pathological brain changes associated with AD is a challenging task, especially considering that people do not show symptoms of *dementia* until late into the disease course. The support of eye-tracking technology opens the possibility of getting closer to the invisible part of neuronal connections, overcoming limitations related to self-reported methodologies (Connors et al., 2016; Bell et al., 2018). Therefore, eye-trackers are powerful precision instruments ready to accelerate the transition toward a non-invasive and accessible screening procedure for MCI. As outlined in this review, eye-tracking technology can be useful in detecting early signs of decline in combination with experimental paradigms investigating cognitive function including memory loss and difficulties with attention and processing speed.

Eye-movement-based cognitive scoring is an area of active research and development, with ongoing studies aiming to refine and improve the accuracy and reliability of used tools. Experimental paradigms described in this work provide a promising direction for gaze parameters serving as potential biomarkers to assess symptoms of cognitive decline, with the ultimate goal of indicating the preclinical stages of AD (Crutcher et al., 2009; Zola et al., 2013). However, due to methodological differences in applied paradigms, selection of subjects, choice of the apparatus, and length of follow-up in longitudinal studies, discrepancies between results of the studies may occur.

Of particular importance is the assessment of methodological frameworks and transparent reporting. Notably, while implementing pro- and antisaccade tasks, one should consider that disparity in carried-out conditions (gap or overlap) may account for ambiguity in the findings and, as a direct consequence, the selection of parameters relevant in distinguishing between MCI subtypes, AD patients, and HC. The “gap” effect, for instance, may account for a change in participants’ saccade latencies (Polden et al., 2020) and, as a result, yield conflicting findings. Moreover, difficulty in disengaging attention from the fixation dot presented in the center of the screen would account for slowing down in prosaccade task.

Regarding the instruction-less paradigm methodology, such protocols can be useful in assessing the cognitive capacities of older adults, especially those who have problems with language comprehension. The absence of an explicit instruction may remove any influences that would dictate where participants should direct their gaze. On the other hand, it has been suggested that an increased level of complexity of goal-directed eye movement tasks may be required to robustly identify preclinical stages of cognitive impairment.

Eye-tracking-based cognitive screening tools being investigated and replicated across various populations is another crucial aspect to be addressed. Since demographic and ethnic differences have been identified as influencing eye movement patterns, it is important to take these factors into account when interpreting gaze behavior data. As an illustrative example, we use two studies (Kawagoe et al., 2017; McCade et al., 2018) that both used photographs of human faces in their experimental protocols. While Kawagoe and colleagues observed face-specific abnormalities in scanning behavior in the aMCI group, McCade and colleagues reported comparable face scanning behavior among all three groups (aMCI, naMCI, and HCs). In addition, given that facial processing deficits may appear in various clinical populations (including AD, aMC, depressive disorder, and schizophrenia), it may seem challenging to differentiate between different clinical entities while following a face recognition task. In order to differentiate between healthy aging adults and patients suffering from disorders, scrupulous comparison of clinical subtypes across various populations is important. Reports of such studies may support the choice of the most promising set of gaze metrics as future biomarkers for AD-related MCI, increasing the opportunities for early intervention.

In 2020, Lehtola and colleagues investigated whether computer-based eye-tracking analysis of the KD test could differentiate patients with idiopathic normal pressure hydrocephalus (iNPH; a progressive neurodegenerative disease with characteristic symptoms of gait disturbance, cognitive decline, and urinary incontinence) from cognitively unimpaired adults and individuals with AD. The research group followed previous statements that the combination of eye-tracking technology and the KD test constitute an easy-to-use test battery to differentiate disorders characterized by memory impairments (Galetta et al., 2017; Hannonen et al., 2022). However, although the tested parameters (total time used for the reading test, number of errors, durations of fixation and saccade, and saccade amplitudes) significantly differed between the AD group and the cognitively unimpaired group, no significant differences between the patients with iNPH and AD group were detected. Accordingly, extensive investigations are needed to test the possibility of gaze metrics to distinguish AD from other disorders or diseases. In this regard, machine learning methods could analyze scores from a combination of psychological and eye movement tests to predict the trajectory of an individual’s AD progression (Haque et al., 2021; Thabtah et al., 2022a).

### 7.2. … on innovation as integral part of the MCI screening process

In recent years, several research groups showed that deep-learning models combined with eye-tracking technology have good performance in identifying neurological diseases. For example, Chaabouni and colleagues developed a deep-learning architecture to predict the visual attention model of patients with dementia and reached a predictive accuracy of 99.27% (Chaabouni et al., 2017). Furthermore, Biondi and colleagues developed a deep-learning approach to differentiate between the reading behavior of patients with AD and healthy controls. Notably, their presented model had 89.78% accuracy for identifying the cognitively impaired AD group (Biondi et al., 2018). Therefore, it should not pass unnoticed that insights on gaze parameters such as fixations, saccades, and regions/areas of interest provide valuable information for developing eye-tracking-based cognitive tests (Bott et al., 2017; Gills et al., 2019; Oyama et al., 2019; Tadokoro et al., 2021). However, the lack of large-scale eye-tracking datasets is a limiting factor for using deep-learning models for the recognition or classification of AD-related MCI based on eye movement data. Therefore, it is important for the scientific community to establish access to such databases in order to advance the development of machine-learning and deep-learning-based models for identifying cognitive function impairment with higher sensitivity (Fabrizio et al., 2021; Haque et al., 2021; Miltiadous et al., 2021; Rutkowski et al., 2021; Rizzo et al., 2022; Sun et al., 2022). While remarkable advancements are occurring in the field of digital health sector (Dagum, 2018; Kourtis et al., 2019; Topol, 2019; Khan and Javaid, 2021), the challenge is for healthcare system leaders to stay abreast of the latest findings and information about gaze metrics as an emerging option for cognitive screening. Therefore, this systematic review should provide a comprehensive overview of the latest evidence-based knowledge and establish a basis for further advancements.

### 7.3. Limitations

A limitation of any review is the possibility that relevant studies may have not been identified due to the selection of databases and search strings used. In order to reduce the likelihood of omitting relevant papers, reference lists of all studies included in this work were additionally screened. Following the aim to provide a quality review on future biological markers for AD, it is important to underline that the authors focused primarily on paradigms that compared visual information processes between older adults with aMCI and their age-matched control group. Notably, while the review covers various paradigms, the number of representative studies is limited. This observation should be considered by interdisciplinary research groups when proposing follow-up and/or alternative paradigms for assessing cognitive functioning among older adults.

### 7.4. Conclusion

The findings of this systematic review have indicated that eye-tracking-based paradigms may ameliorate the screening limitations of traditional cognitive assessments and contribute to early AD detection. However, before widespread clinical adoption, longitudinal investigations in lab-based and ecologically valid settings are necessary to translate the findings relating to abnormal gaze behavior.

## Data availability statement

The original contributions presented in the study are included in the article/Supplementary material, further inquiries can be directed to the corresponding author.

## Author contributions

AW, KT, and MO-M made the conceptualization and methodology. AW and KT have done identification and screening of the scientific reports. AW done the draft preparation, revisions, and editing. MO-M and SU provided expertise in the field and critically revised the manuscript. All authors contributed to the article and approved the submitted version.

## Funding

This work was supported by the Special Postdoctoral Research (SPDR) grant under the RIKEN SPDR Fellowship (AW), JSPS KAKENHI (Grant Numbers: JP22H04872, JP22H00544) and the Japan Science and Technology Agency (Grant Numbers: JPMJCR20G1, JPMJPF2101, and JPMJMS2237).

## Conflict of interest

The authors declare that the research was conducted in the absence of any commercial or financial relationships that could be construed as a potential conflict of interest.

## Publisher’s note

All claims expressed in this article are solely those of the authors and do not necessarily represent those of their affiliated organizations, or those of the publisher, the editors and the reviewers. Any product that may be evaluated in this article, or claim that may be made by its manufacturer, is not guaranteed or endorsed by the publisher.
